# Antioxidant Activity and ROS-Dependent Apoptotic Effect of *Scurrula ferruginea* (Jack) Danser Methanol Extract in Human Breast Cancer Cell MDA-MB-231

**DOI:** 10.1371/journal.pone.0158942

**Published:** 2016-07-13

**Authors:** Mohsen Marvibaigi, Neda Amini, Eko Supriyanto, Fadzilah Adibah Abdul Majid, Saravana Kumar Jaganathan, Shajarahtunnur Jamil, Javad Hamzehalipour Almaki, Rozita Nasiri

**Affiliations:** 1 IJN-UTM Cardiovascular Engineering Center, Faculty of Biosciences and Medical Engineering, Universiti Teknologi Malaysia, Skudai, Johor, Malaysia; 2 Bioprocess Engineering Department, Faculty of Chemical Engineering, Universiti Teknologi Malaysia, Skudai, Johor, Malaysia; 3 Department of Chemistry, Faculty of Science, Universiti Teknologi Malaysia, UTM Johor Bahru, Johor, Malaysia; 4 Institute of Marine Biotechnology, Universiti Malaysia Terengganu, Kuala Terengganu, Malaysia; Columbia University, UNITED STATES

## Abstract

*Scurrula ferruginea* (Jack) Danser is one of the mistletoe species belonging to Loranthaceae family, which grows on the branches of many deciduous trees in tropical countries. This study evaluated the antioxidant activities of *S*. *ferruginea* extracts. The cytotoxic activity of the selected extracts, which showed potent antioxidant activities, and high phenolic and flavonoid contents, were investigated in human breast cancer cell line (MDA-MB-231) and non-cancer human skin fibroblast cells (HSF-1184). The activities and characteristics varied depending on the different parts of *S*. *ferruginea*, solvent polarity, and concentrations of extracts. The stem methanol extract showed the highest amount of both phenolic (273.51 ± 4.84 mg gallic acid/g extract) and flavonoid contents (163.41 ± 4.62 mg catechin/g extract) and strong DPPH^•^ radical scavenging (IC_50_ = 27.81 μg/mL) and metal chelation activity (IC_50_ = 80.20 μg/mL). The stem aqueous extract showed the highest ABTS^•+^ scavenging ability. The stem methanol and aqueous extracts exhibited dose-dependent cytotoxic activity against MDA-MB-231 cells with IC_50_ of 19.27 and 50.35 μg/mL, respectively. Furthermore, the extracts inhibited the migration and colony formation of MDA-MB-231 cells in a concentration-dependent manner. Morphological observations revealed hallmark properties of apoptosis in treated cells. The methanol extract induced an increase in ROS generation and mitochondrial depolarization in MDA-MB-231 cells, suggesting its potent apoptotic activity. The present study demonstrated that the *S*. *ferruginea* methanol extract mediated MDA-MB-231 cell growth inhibition via induction of apoptosis which was confirmed by Western blot analysis. It may be a potential anticancer agent; however, its *in vivo* anticancer activity needs to be investigated.

## Introduction

Plant-derived antioxidants protect biological systems from oxidative stress generated by free radicals or reactive oxygen species (ROS) during metabolism and other activities. Antioxidants have a preventive role in numerous disorders caused by cellular damage or oxidative injury including cancer, diabetes, cardiovascular diseases, and mutagenesis [1–3]. Secondary metabolites, such as carotenoids, phenols, ascorbic acid, and flavonoids, are potential sources of natural antioxidants with free radical scavenging capacity [4, 5]. The therapeutic potential of medicinal plants is generally attributed to the antioxidant activity of phytochemicals, particularly phenols and flavonoids [6, 7]. Herbal medicines play a key role in the development of new potential drugs. There is a large and growing body of literature on the discovery of secondary metabolites with antioxidant capacity, and new phytochemical constituents (particularly anticancer agents) from various medicinal plants [8]. Costly treatment methods and serious side effects associated with available therapies may lead to greater tendencies of using herbal medicines for health care. Mistletoe is a common semi-parasitic evergreen plant from the flowering plant family Loranthaceae, which comprises approximately 1500 species that grow on branches of many deciduous trees worldwide [9, 10]. Mistletoe is one of the most widely used herbal medicines with a long history of use in the treatment of various disorders, such as diabetes, skin infection, smallpox, and cough. Steiner [11] introduced it in the field of oncology as an alternative therapy for cancer care. For decades, natives of south Asia, Europe, and Africa have extensively used mistletoe as a complementary and alternative medicine in the treatment and management of numerous diseases including cancer. Several studies have shown that mistletoe, as an anthroposophical medicine, is one of the most important medicinal plants that are potentially efficacious against cancer [12, 13]. Numerous preclinical and *in vitro* studies using different commercial and standardized products of mistletoe have reported its immunomodulatory, anti-tumor, and anti-metastatic effects [14–20]. Various mistletoe extracts from different origins are capable of inducing apoptosis and cell death in numerous types of tumors and human cancer cell lines [21, 22]. The majority of the studies conducted by the European researchers, particularly investigators from Germany, employed *Viscum album* (European mistletoe). Extensive preclinical and clinical investigations have been carried out on European mistletoe. However, species of mistletoe from other continents have not received much attention. One such mistletoe species belonging to Loranthaceae family is *Scurrula ferruginea*, which is mainly distributed in tropical countries [23]. It is locally known as Dedalu in Malaysia, Singapore, and Indonesia. The plant is used traditionally to treat various diseases including gastrointestinal malfunction, high blood pressure, hypertension, and malaria [24–26]. For example, Ameer *et al*. [24] investigated the effect of *S*. *ferruginea* extracts on blood pressure by using *in vitro* and *in vivo* animal experimental models. They demonstrated the presence of biologically active substances in *S*. *ferruginea* and found that the methanol extract possessed the highest blood pressure lowering activity. They attributed it to the high content of phenols and flavonoids in this plant. Their results provided direct evidence of blood pressure lowering activity of *S*. *ferruginea*. Another study [27] evaluated the cytotoxic effects of *S*. *ferruginea* extract on different human cancer cell lines. The main constituents of the ethyl acetate fraction including quercetin, quercitrin, and glycoside 4-O acetylquercitrin were isolated using column chromatography. Quercetin exhibited the most potent cytotoxic activity against U251 (human glioblastoma cell line) cells with an IC_50_ of 35μM. *S*. *ferruginea* has been used in traditional medicine for the management of various diseases. Some studies have also reported its therapeutic potential. However, to our knowledge, the antioxidant activity of methanolic, aqueous, ethyl acetate, and hexane extracts from various parts of *S*. *ferruginea* have not been explored. Therefore, the first objective of the present study was to screen for the antioxidant activity of different extracts from stem, leaves, and flowers of *S*. *ferruginea in vitro*. *In vitro* assays were carried out for determination of DPPH free radical (DPPH^•^) scavenging capacity, total phenolic content (TPC), total flavonoid content (TFC), metal chelation capacity, and on 2,2ʹ-azinobis-(3-ethyl-benzothiazoline-6-sulfonic acid cationic radicals (ABTS^•+^) scavenging ability. The crude extracts exhibiting potential antioxidant activity, and high total phenolic and flavonoid contents were selected for further investigation of anticancer and apoptotic activity. Only one study, which focused exclusively on MCF-7 cells, has investigated the anticancer activity of *S*. *ferruginea* [28]. The effect of *S*. *ferruginea* extracts on claudin-low or triple-negative breast cancer cells (MDA-MB-231), which is the most aggressive subtype of breast cancer with poor prognosis, has not been previously reported. Additionally, its effect on non-cancerous cell line (HSF-1184) has not been investigated. Furthermore, the proapoptotic activity and antimetastatic potential of *S*. *ferruginea* in MDA-MB-231 cells have not been investigated. Therefore, the present study was carried out to investigate the proapoptotic activity of *S*. *ferruginea* extracts. Production of ROS and mitochondrial dysfunction were evaluated to investigate the involvement of mitochondria in cell death process after *S*. *ferruginea* treatment. To the best of our knowledge, this is the first to report that the administration of *S*. *ferruginea* methanol extract caused morphological alterations, mitochondrial disruption, ROS generation, and apoptosis induction in MDA-MB-231 cell.

## Materials and Methods

### Chemicals and reagents

All chemicals and reagents used in the study including solvents were of analytical grade. Methanol, hexane, and ethyl acetate were obtained from Score Scientific SDN BHD. 1,1-Diphenyl-2-picryl hydrazine (DPPH), 2, 2'-azino bis-(3-ethyl benzo thiazoline-6-sulphonic acid) (ABTS), catechin, Folin Ciocalteu’s phenol reagent, gallic acid, 6-hydroxy-2,5,7,8-tetramethylchroman-2-carboxylic acid (Trolox), sodium nitrite, sodium hydroxide (NaOH), 3-(2-pyridyl)-5,6-diphenyl-1,2,4-triazine-p,p′-disulphonic acid monosodium salt (Ferrozine), sodium carbonate (Na_2_CO_3_), aluminum chloride (AlCl_3_), ferrous sulfate (FeSO_4_), iron (II) chloride (FeCl_2_), iron (III) chloride (FeCl_3_), potassium persulfate (K_2_S_2_O_8_), ethylenediaminetetraacetic acid (EDTA), and ascorbic acid were purchased from Sigma Chemicals (USA). MitoProbe JC-1 assay kit, phosphate buffer saline (PBS), trypsin, penicillin, DMEM (Dulbecco’s modified Eagle medium), and fetal bovine serum (FBS) were obtained from Life Technologies Inc., Grand Island, NY, USA. Hoechst 33342, tamoxifen, propidium iodide (PI), 2′,7′-dichlorofluorescein diacetate, acridine orang, and ethidium bromide were purchased from Sigma-Aldrich, USA. Dimethyl sulfoxide (DMSO) was obtained from RCI Labscan (Thailand). Human breast cancer cell line (MDA-MB-231, ATCC^®^ HTB-26^™^) and human skin fibroblast cell line (HSF-1184, ATCC^®^ PCS-201-012^™^) were purchased from the American Type Culture Collection (ATCC). MTT [3-(4,5-dimethylthiazol-2-yl)-2,5-diphenyltetrazolium bromide] was obtained from Invitrogen. All primary antibodies including Bax (6A7), Bcl-2 (124), PARP (C-2-10), caspase-3, caspase-7 (B4-G2), goat anti-rabbit IgG-AP secondary antibody, and goat anti-mouse IgG (H+L) secondary antibody alkaline phosphatase were purchased from Pierce. β-Actin (13E5) rabbit mAb primary antibody was taken from Cell Signaling Technology.

### Plant material

Aerial parts of *S*. *ferruginea* (Jack) Danser were collected from the campus of Universiti Teknologi Malaysia (Latitude N 1° 33’ 54.9", Longitude E 103° 38’ 29.2"), Skudai, Johor, Malaysia in September 2012 and separated to leaves, stems, and flowers. The plant was authenticated and taxonomically identified by Dr. Shamsul Khamis of biodiversity unit, Institute of Bioscience, Universiti Putra Malaysia (UPM). A voucher specimen (SK 2529/14) was deposited at the same institute.

### Extraction of *S*. *ferruginea* leaves, stems, and flowers

The plant materials were cleaned of any extraneous materials using tap water followed by distilled water. They were then air-dried in shade at 30 ± 2°C and pulverized into powder. The extracts were prepared as previously described [29] with slight modifications. The powdered plant materials (50 g) were sequentially macerated with hexane, ethyl acetate, methanol, and water (500 mL each) in a platform shaker for three days at room temperature. Solutions obtained after each maceration step were filtered using Whatman^®^ filter paper. The whole extraction procedure was repeated 3 times, each time using fresh solvents. The filtrates were pooled together and fully evaporated using a rotary evaporator (BUCHI, Switzerland, and R210) in a water bath at 50°C. The obtained aqueous extracts were freeze dried while the methanol, ethyl acetate, and hexane extracts were oven-dried at 40°C. Finally, the obtained extracts were weighed, their yield was calculated, and stored at -20°C in sealed tubes until used.

### Estimation of total phenolic content (TPC)

The total phenolic contents of *S*. *ferruginea* extracts were determined by Folin-Ciocalteu colorimetric method as reported earlier [30], with slight modifications. Plant extracts (200 μL) were added to 1 mL of Folin-Ciocalteu reagent (diluted 1:10 in deionized distilled water). The mixtures were allowed to stand at room temperature for 5 minutes with intermittent shaking. Subsequently, 800 μL 7.5% Na_2_CO_3_ was added and the mixture was incubated for 30 minutes at room temperature in dark. The absorbance (of the resulting blue color) was measured at 760 nm on a spectrophotometer and compared to gallic acid (used as a standard) calibration curve to determine the TPC. All TPC determinations were carried out three times (n = 3) and the results were expressed as mg gallic acid/g of dry extract.

### Determination of total flavonoid content (TFC)

The total flavonoid content was measured spectrophotometrically by aluminum chloride colorimetric method, which is based on the formation of a flavonoid–aluminum complex [31]. Plant extract (1mL of1 mg/mL) was added to a 10 mL volumetric flask and mixed with 300 μl 5% NaNO_2_. The mixture was incubated for 6 minutes at ambient temperature. Subsequently, 300 μl 10% AlCl_3_ was added to the flask. After another 6 minutes of incubation, 1.5 mL of 4.3% NaOH was added and the final volume of reaction mixture was brought up to 10 mL with distilled water. The absorbance of samples was read at 515 nm using UV spectrophotometer and compared with catechin (50–250 μg/mL) calibration curve, which was made by treating catechin using the same procedure. Catechin was utilized as a standard and the TFC of extracts was expressed in milligram catechin/gram extract. The analysis was performed in triplicate and mean values were reported.

### DPPH radical scavenging capacity free

Free radical scavenging potential of the *S*. *ferruginea* extracts was measured based on their capability to scavenge the stable DPPH^•^ according to a previously described method [32] with some modifications. Working concentrations of extracts (7.81–1000 μg/mL) were prepared from the stock solutions (2 mg/mL) of extracts (of different *S*. *ferruginea* parts) in methanol. A 0.2 mM DPPH solution was prepared in methanol. An aliquot (100 μL) of each extract working concentration was added to 100 μL of DPPH. The mixtures were shaken thoroughly and allowed to stand in dark at room temperature for 30 minutes. A color change from violet to yellow occurred during the reaction time. Sample absorbance was read at 517 nm using UV-VIS spectrophotometer and the percentage of free radical scavenging potential of the different extracts against DPPH^•^ was determined using the following equation.

$$\%\;\text{Inhibition}=\left(\frac{\text{Control absorbance}-\text{Sample absorbance}}{\text{Control absorbance}}\right)\times 100$$

DPPH solution was used as a control and ascorbic acid (Vitamin C) was used as a reference standard. IC_50_, the concentration of sample required to reduce 50% DPPH^•^, was calculated for all samples. All samples were assayed in triplicate.

### Ferrous ion-chelating capacity assay

The Fe^2+^ chelating activity of *S*. *ferruginea* extracts was determined by a previously reported method [33], which is based on the formation of the Fe^2+^-ferrozine complex, with slight modifications. Briefly, 20 μL of 1 mM FeCl_2_ (ferrous chloride) was added to different concentrations (2000, 1000, 500, 250, 125, 62.5 μg/mL) of samples. Subsequently, 60 μL of 3 mM ferrozine was added to the mixture to initiate the reaction of ferrozine with divalent iron. This reaction resulted in the formation of stable and water-soluble magenta colored complexes. After incubation at room temperature, the absorbance was measured at 570 nm. EDTA and distilled water (instead of ferrozine) were used as standard and blank, respectively. All determinations were carried out in triplicate. The Fe^2+^-chelating ability of the extracts was calculated using the following equation where AC represents the absorbance of control and AS is the absorbance of reaction mixture in presence of extracts or EDTA.

$$\text{\% Inhibition of ferrozin}-\text{iron complex formation}=\left(\frac{\text{AC}-\text{AS}}{\text{AC}}\right)\times 100$$

### ABTS radical scavenging activity of *S*. *ferruginea* extracts by TEAC method

The free radical scavenging capacity of *S*. *ferruginea* extracts was also measured using TEAC (Trolox equivalent antioxidant capacity) method [34]. This spectrophotometric assay evaluates the ability of substances to scavenge the ABTS^•+^ in relation to Trolox. ABTS^•+^ was produced by reacting 7.4 mM ABTS and 2.45 mM potassium persulfate stock solutions in equal quantities at room temperature for 16 h in dark. The ABTS^•+^ stock solution was diluted with distilled water to obtain an absorbance of 0.70 ± 0.02 units at 734 nm. In order to study the antioxidant activity of the extracts, freshly prepared ABTS^•+^ solution (300 μL) was added to 30 μL of the extracts and allowed to react for 10 minutes in dark. Subsequently, the absorbance was measured at 734 nm. A standard curve was obtained from different concentrations of Trolox standard solution. All experiments were performed in triplicate and the results were expressed in μM Trolox/g extract.

### Cell culture

MDA-MB-231 breast cancer cells were used for determination of cytotoxicity. HSF-1184 cell line was used as control. MDA-MB-231 and HSF-1184 cells were maintained as monolayer cultures in DMEM supplemented with 10% FBS and 1% penicillin-streptomycin (100 units/mL-100 μg/mL) respectively. Adherent cell monolayers were cultured in T25 tissue culture flasks for passage and experiments. Both cell lines were maintained at 37°C in a humidified 5% carbon dioxide (CO_2_) atmosphere.

### Cell proliferation assay

The *in vitro* cytotoxic activities of *S*. *ferruginea* extracts were evaluated using MTT assay [35]. Cells were trypsinized and counted by dye exclusion assay using hemocytometer (FORTUNA, Germany). Cells were seeded in 96-well plates at a density of 1×10^5^ cells/well in 200 μL DMEM overnight. Cells were then treated with different concentrations of the extracts for 24, 48, and 72 h. After the treatment, 20 μL of MTT reagent was added to each well, and the plates were then incubated for 4 h at 37°C. Subsequently, the medium containing MTT was aspirated and the formazan crystals were solubilized by the addition of 200 μL DMSO. The absorbance of colored formazan product was measured at 570 nm using ELISA microplate reader. Tamoxifen and DMSO (0.1%) were used as positive reference standard and negative control, respectively. The experiments were performed in triplicate. The percentage of cell viability was calculated as the ratio of sample absorbance to control absorbance (0.1% DMSO).

$$\text{\% Cell viability}=\left(\frac{\text{Absorbance of treated cells}}{\text{Absorbance of control }\left(\text{DMSO}\right)}\right)\times 100$$

### Morphological observation of MDA-MB-231 cells

Morphology of MDA-MB-231 cells treated with or without *S*. *ferruginea* extracts was compared to determine the changes induced by the extracts. MDA-MB-231 cells were plated in 12-well plates and exposed to increasing concentrations (31.25 to 1000 μg/mL) of *S*. *ferruginea* extracts for 24, 48, and 72 h. The morphological changes were observed using inverted phase contrast microscope (Zeiss Axiovert 100) at 20 X magnification.

### Ethidium bromide and acridine orange staining for apoptosis detection

Early apoptotic, late apoptotic, and necrotic cells can be detected using fluorescent staining methods. Here, acridine orange (AO) and ethidium bromide (EB) staining method was used to investigate the induction of apoptosis by *S*. *ferruginea* extracts. MDA-MB-231 cells were seeded in 24-well plates. Confluent cell monolayers were exposed to various concentrations of the extracts. Treatments were carried out for 24 h at 37°C following which the cells were gently washed with PBS. Cells were then immediately treated with a dye mixture containing EB (100 μg/mL) and AO (100 μg/mL) in a 1:1 ratio for 5 minutes. Subsequently, cells were imaged using a florescence microscope (ZEISS AXIOVERT A1) equipped with a digital imaging system. AO is a cell-permeant dye and stains the nuclei green in living cells. In contrast, EB can only permeate dead cells or in cells with ruptured plasma membranes and stains the nucleus orange. Therefore, live cells show green nuclei with organized structure and the dead or necrotic cells display bright orange chromatin in round nuclei. Early apoptotic cells exhibit bright green/yellowish fragmented and condensed chromatin. Late apoptotic cells display orange nuclei with condensed and fragmented DNA.

The assay was performed in triplicate. A minimum of 200 total target cells were counted and the number of viable, apoptotic, and necrotic cells as indicated by the nuclear morphology and membrane integrity was recorded and analyzed. The percentages of apoptotic and necrotic cells were then calculated using the following formulae:

$$\text{Percentage of apoptotic cells }\left(\text{\%}\right)=\left(\frac{\text{total number of early and}/\text{late apoptotic cells}}{\text{total number of cells }}\right)\times 100$$

$$\text{Percentage of necrotic cells }\left(\text{\%}\right)=\left(\frac{\text{total number of necrotic cells}}{\text{total number of cells }}\;\right)\times 100$$

### Propidium iodide and Hoechst 33342 staining for study of nuclear morphology

The apoptotic morphology of cells, such as nuclear shrinkage and chromatin condensation, can be observed under fluorescence microscope after nuclear staining with PI and Hoechst 33342 (DNA binding dyes). Fluorescence staining of nuclei was carried out to further confirm whether the aqueous and methanol extracts induced inhibition of MDA-MB-231 cell growth via apoptosis. MDA-MB-231 cells were seeded and treated with different concentrations of the methanol and aqueous extracts for 24 h at 37°C and 5% CO_2_. After treatment with the extracts, the cells were washed with PBS and fixed in 4% paraformaldehyde (methanol free) for 30 minutes. Subsequently, cells were washed again with PBS followed by staining with Hoechst 33342 (10 μg/mL) and PI (10 μg/mL), respectively. Cells were then incubated in dark at room temperature for 30 minutes. Lastly, the cells were washed again with PBS and analyzed under an inverted fluorescence microscope (ZEISS AXIOVERT A1).

### Clonogenic inhibition assay

Clonogenic cell survival assay was carried out as described previously [36] with some modifications. Briefly, cells in log phase of growth were trypsinized and approximately 1*×*10^3^ cells/mL were seeded into six-well plates in triplicate. The next day, exhausted medium was changed with fresh medium containing different concentrations (1000, 500, 250, 125, 62.5, 31.25 μg/mL) of the *S*. *ferruginea* extracts. Treatment was carried out for 24 h following which. The cells were then cultured for 14 days with growth medium replaced every two days. On day 15, the resulting colonies were fixed and stained with 0.5% trypan blue solution. Only colonies with 50 or more cells were counted and expressed as a percentage of untreated control cultures.

### *In vitro* scratch motility assay

In order to investigate the cell migration inhibition efficiency of the extracts, the wound healing assay was performed in MDA-MB-231 cells as described in [37] with some modifications. Briefly, cells (3x10^5^ cells per well) were seeded in a 6-well plate and allowed to grow overnight at 37°C and 5% CO_2_ to form a confluent monolayer. After 24 h of serum starvation, linear ‘scratch’ wounds were created on the monolayer using a sterile cell scrapper. The cells were washed gently with PBS twice to remove cellular debris and detached cells. Subsequently, the cells were treated with the extracts (31.25 to 1000 μg/mL). The cells in negative control wells were incubated with complete culture medium. The plates were incubated at 37°C and the wound was photographed at 0, 12, and 24 h. At each time point, the distance over which the cells migrated was measured. Photographs were captured using an inverted phase contrast microscope (Zeiss Axiovert 100) equipped with a digital camera and the data were analyzed using NIH Image J software. Wound closure rates were then calculated quantitatively as the difference between wound width at 0, 6, and 12 or 24 h. The results were expressed as percentage cell migration. Three independent experiments were performed for each sample.

### Determination of intracellular ROS generation

Intracellular ROS levels of living cells were measured quantitatively and qualitatively by using a fluorescent probe, 2′,7′-dichlorodihydrofluorescein diacetate (DCF-DA). DCF-DA is an oxidation-sensitive fluorescent probe that is cleaved by intracellular esterases. It is rapidly oxidized to the highly fluorescent DCF (2′,7′- dichlorofluorescein), which can be readily detected by spectrofluorometer, in the presence of intracellular hydrogen peroxide and peroxidases [38, 39]. MDA-MB-231 cells were treated with various concentrations (31.25–1000μg/mL) of the methanol extract for 12 h. Treated or untreated cells were incubated with 20 μM DCF-DA for 45 minutes at 37°C in dark, and subsequently washed with PBS. Relative changes in intracellular ROS levels were monitored by fluorometric detection of DCF using a fluorescent microplate reader (GloMax^®^-Multi Detection System, Promega, USA) at excitation and emission wavelengths of 485 nm and 530 nm, respectively. The fluorescence intensity of DCF is proportional to the level of ROS generated intracellularly. The fluorescence was also monitored qualitatively by an inverted fluorescent microscope (Zeiss Axiovert A1) at an excitation and emission wavelength of 488 nm and 560 nm, respectively.

### Mitochondrial membrane potential assay (JC-1 assay)

The mitochondria-specific cationic fluorescence dye JC-1 (MitoProbe^™^ JC-1 Assay Kit) was used to determine the mitochondrial membrane potential (MMP). JC-1 (5,5',6,6'-tetrachloro-1,1'3,3'- tetraethyl benzamidazol-carbocyanine iodide) is a lipophilic dye that undergoes potential dependent accumulation in mitochondria. It is a dual-emission fluorescent dye capable of selectively entering the mitochondria. It can reversibly change its color from red to green when the membrane potential is decreased. In healthy cells with normal polarized mitochondria, JC- 1 forms aggregates and emits intense red fluorescence. However, it forms monomers that show green fluorescence in unhealthy or apoptotic cells with depolarized mitochondrial membranes. The MMP depolarization is one of the early key events in apoptosis [40, 41]. The mitochondrial depolarization patterns of the cells were measured using the JC-1 dye both qualitatively and quantitatively. MDA-MB-231 cells were seeded in 96-well culture plates at a density of 5×10^4^ cells/well. After 12 h incubation, cells were treated with various concentrations of methanol extract (31.25–1000μg/mL). Treatment was carried out for 24 h following which the culture medium was removed and the cells were stained with 2 μM JC-1 (in PBS) for 45 minutes at 37°C. Subsequently, cells were washed with PBS, and observed using an inverted fluorescent microscope (Zeiss Axiovert A1, magnification: 40x) for qualitative analysis of JC-1 uptake by mitochondria. Quantitative assessment of JC-1 uptake was performed using a fluorescence plate reader (GloMax^®^-Multi Detection System, Promega, USA). It detects JC-1 aggregates at excitation/emission wavelengths of 540/570 nm and JC-monomers at excitation/emission wavelengths of 485/535 nm. The percentage of cells showing MMP changes was calculated from triplicate. The results were represented as ratio of red to green fluorescence compared with the control.

### Western blotting

MDA-MB-231 cells cultured in T75 flasks to 80% confluence and were treated with methanolic extracts of *S*. *ferruginea* at IC_50_ for 0, 2, 4, 6, 12, and 24 h. After treatment with the extract, cells were harvested and rinsed with ice-cold PBS. Subsequently, the cells were lysed with Complete^™^ lysis–M reagent (Roche Diagnostics GmbH, Mannheim, Germany), containing an EDTA-free efficient lysis reagent and protease inhibitor cocktail. Lysed cells were then scraped using a cold cell scraper, and centrifuged at 14000 × g for 10 minutes at 4°C. Supernatant (total cell protein) was collected and protein concentration was measured using BCA assay (Bicinchoninic Acid) according to the Pierce^™^ BCA Protein Assay Kit (Thermo Scientific). Equal amounts of protein from each lysate were resolved on 12.5% SDS–PAGE gels (w/v). The SDS-PAGE was performed using a Biorad Mini-PROTEAN ll electrophoresis system (Bio-Rad, Hercules, CA, USA). The resolved proteins were transferred to Immun-Blot PVDF transfer membrane (BIO-RAD) at 100 V and 350 mA for 1 h. The primary antibodies were then prepared and incubated with the membrane at 4°C overnight. Subsequently, the membrane was washed with TBST (1 × TBS, 0.1% Tween-20) three times for 10 minutes. The membrane was then incubated with alkaline phosphatase (AP)-conjugated goat anti-mouse IgG secondary antibody for 1 h at room temperature under agitation. Thereafter, the membrane was washed five times with TBST. BCIP/NBT (5-bromo-4-chloro-3-indolyl phosphate/nitro blue tetrazolium) substrate was used for colorimetric AP detection. The colorimetric detection of AP is based on the reaction of substrate with alkaline phosphatase (AP) bound to the secondary antibody. This reaction converts the soluble dye into an insoluble form, which produces a colored precipitate, thus allowing visualization of the protein on a Western blot. β-Actin was used in each blot as a loading control. Densitometric analysis and protein quantification was carried out using ImageJ software. The intensity of bands from control and treated samples was measured, and the relative level of proteins was normalized to β-actin.

### Statistical analysis

Results of all analysis are presented as means of triplicate ± standard deviation (SD) (n = 3). Non-linear regression analysis was performed using GraphPad Prism statistical software to calculate the IC_50_ and respective 95% confidence intervals (95% CI) for antioxidant and cytotoxic activities. The overall effects of plant part (P), solvent type (S), and the interaction (P × S) on phenolic and flavonoid contents, and ABTS^•+^ scavenging capacity were determined using two-factor analysis of variance (ANOVA). Two-factor ANOVA with type III sums of squares was performed using the general linear model (GLM) procedure in SPSS (version 21.0). Bonferroni post hoc test was used to check for any significant differences between data. Three-factor ANOVA and post hoc multiple comparison was carried out to analyze the influence of P, S, concentration (C), and the interaction (P × S × C) on DPPH^•^ scavenging capacity and metal chelation activity. The results were considered statistically significant at *P* value *<* 0.05. Pearson correlation coefficient (*r*) was used find the relationships between the total phenolic and flavonoid contents and various antioxidant assays. *p* < 0.05 was considered significant. The cell culture data were subjected to one-way ANOVA followed by Tukey’s multiple comparisons post hoc test. *P* value <0.05 was considered statistically significant.

## Results

### Extraction yields

The weight percentage of the obtained dried crude extract with respect to the initial amount of the dried powder is defined as yield (Table 1). Considerable variations in the percentage yields were found among the extracts obtained using different solvents. The lowest percentage yield was recorded for flower hexane extract, while the highest yield was observed in the leaf methanol extract. Large differences were observed between extraction yields of methanol and hexane extracts for all parts of plant. Hexane was the least polar solvent, which results in lower extraction yield than others solvents. Analysis of the total amount of crude extract obtained from different parts of *S*. *ferruginea* showed that the leaf extracts had the highest yield followed by the stem and flower extracts. Higher yield of stem, flower, and leaf methanol extracts could be attributed to high vapor pressure of methanol.

**Table 1:** Percentage extraction yields obtained from different *S*. *ferruginea* parts using different solvents.

		Yields (%)		
Extract	Water	Methanol	Ethyl acetate	Hexane
**Stem**	17.34 ± 0.33	20.38 ± 0.31	11.79 ± 0.19	8.58 ± 0.43
**Leaf**	19.59 ± 0.20	22.39 ± 0.22	13.67 ± 0.31	9.48 ± 0.15
**Flower**	16.09 ± 0.23	18.72 ± 0.17	10.79 ± 0.13	7.63 ± 0.22

All results are means ± SD (n = 3) of triplicate determinations.

### TPC of *S*. *ferruginea* extracts

Table 2 summarizes the TPC in stem, leaf, and flower *S*. *ferruginea* extracts. Two-way ANOVA revealed that S, P, and S × P had significant effects (P < 0.05) on TPC. These analyses indicated that both S and P influenced the TPC of extracts. Results of TPC showed significant differences between stem, flower, and leaf as well as between different extracting solvents (P < 0.05). The stem methanolic extract had significantly higher phenolic content than others (P < 0.05). The TPC of methanol and aqueous extracts of all plant parts were statistically different from each other (*P <*0.05). However, there were no significant differences in the phenolic contents of the stem and leaf hexane and ethyl acetate extracts (P > 0.05). The lowest TPC (4.34±1.72 mg gallic acid/g) was observed for flower hexane extract, which also showed significant differences from the extracts of other solvents and parts (*p* < 0.05). The TPC results indicated that a higher solvent polarity yields higher amounts of phenolic compounds. In other words, results suggested that extraction with low polarity solvents resulted in a lower content of polyphenols.

**Table 2:** TPC of extracts of *S*. *ferruginea* leaves, stems, and flowers using different solvents expressed in mg gallic acid/g extract.

Total phenolic content				
	Solvents			
Plant part	Water	Methanol	Ethyl acetate	Hexane
**Stem**	206.24 ± 8.66^a^	273.51 ± 4.84^b^	35.86 ± 6.88^c^	10.60 ± 2.43^d^
**Leaf**	117.54 ± 2.41^e^	163.29 ± 6.79^f^	29.51 ± 4.45^c^	14.24 ± 3.17^d^*
**Flower**	93.61 ± 6.30^g^	123.05 ± 7.82^h^	18.87 ± 3.21^i^	4.34 ± 1.72^d^*

Results are means ± SD of triplicate from 3 independent experiments. Values with the same superscripts in different groups imply statistical non-significance *P* > 0.05. Same superscripts with asterisk imply statistical significance *P* < 0.05.

### Determination of TFC

The flavonoid content varied from 16.23±1.04 to 163.41±4.62 mg catechin/ g extract (Table 3). Two-way ANOVA demonstrated a statistically significant interaction between S and P (S × P) (P < 0.05). The results revealed that there were significant differences between the stem and leaf extracts as well as between the stem and flower extracts (*P <*0.05). However, there was no significant difference in the flavonoid content of leaf and flower extracts (P > 0.05). The stem methanol extract showed the highest flavonoid content. However, there was no significant difference in TFC between the stem methanolic and aqueous extracts (P > 0.05). Similar to TPC, the lowest flavonoid content was recorded in the flower hexane extracts, which showed no significant difference with the leaf hexane extract (P > 0.05). The TFC is also influenced by the polarities of solvent and plant parts.

**Table 3:** Total flavonoid content of *S*. *ferruginea* leaves, stems, and flowers using different solvents expressed in mg catechin/ g extract.

		Total flavonoids		
		Extractants		
Plant part	Water	Methanol	Ethyl acetate	Hexane
**Stem**	157.17 ± 5.81^ax^	163.41 ± 4.62^ax^	52.85 ± 3.16^bx^	24.64 ± 2.51^cx^*
**Leaf**	86.59 ± 7.07^ay^	70.67 ± 2.49^by^	38.92 ± 7.13^cy^	18.64 ± 3.95^dx^
**Flower**	59.48 ± 4.78^az^	107.60 ± 7.58^bz^	28.00 ± 3.98^cz^	16.23 ± 1.04^dx^*

All analysis were performed in triplicate and expressed as means ± SD. For the same extract with different part of plant, means in the same column with different letters (x-z) were significantly different (*p* < 0.05, two-way ANOVA). For different extracts with the same part of plant, means in the same row with different letters (a-d) were significantly different (*p* < 0.05, two-way ANOVA). Same superscripts with asterisk imply statistical significance (*P* < 0.05).

### DPPH scavenging activity

**Fig 1:**
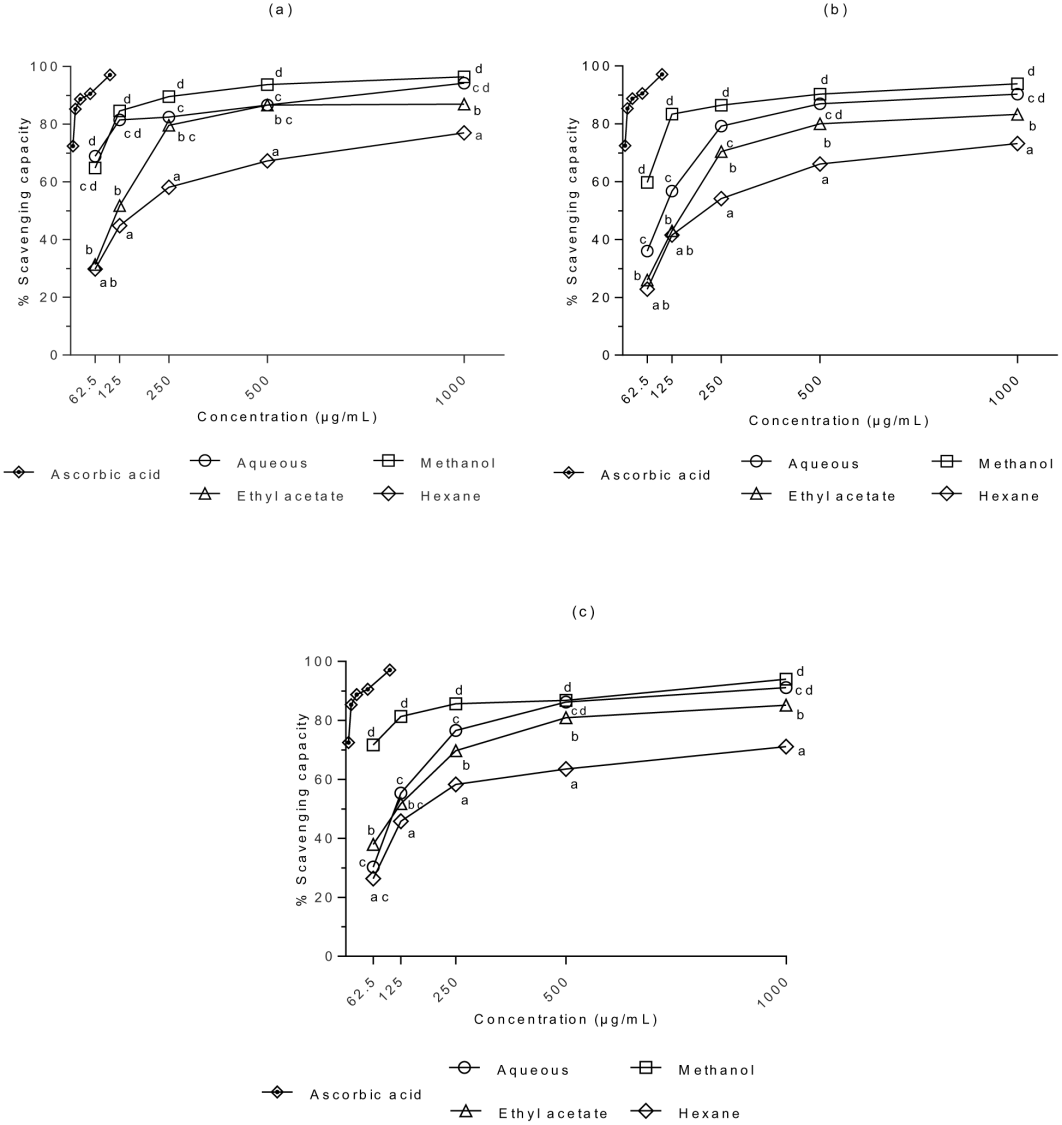
DPPH^•^ scavenging capacities of (a) stem, (b) leaf, and (c) flower extracts of *S*. *ferruginea* and positive control (ascorbic acid). Results are means ± SD (n = 3). Points marked with different letters are significantly different at *P* < 0.05 when compared at the same concentration as determined by two-way ANOVA. The positive control (ascorbic acid) showed a significantly higher scavenging capacity as compared to the samples (*P* < 0.05).

The DPPH^•^ scavenging activities of *S*. *ferruginea* extracts and the positive control are presented in Fig 1. Based on three-factor ANOVA, the effects of S, P, C, and S × P × C on the DPPH^•^ scavenging activity of extracts were statistically significant (p < 0.05). There was a significant difference in DPPH^•^ scavenging activity between the stem and leaf extracts, and between the stem and flower extracts (*P <*0.05). In contrast, no significant differences were observed between the leaf and flower extracts (P > 0.05). The positive control (ascorbic acid) showed a significantly higher scavenging effect than the samples (*P* < 0.05). The stem methanol extract exhibited a potent scavenging effect. However, there was no significant difference between the stem methanol and aqueous extracts (P > 0.05). Generally, the methanol extracts (irrespective of the plant parts or concentrations) exhibited strong antioxidant activity. DPPH color was quenched in a concentration dependent manner by *S*. *ferruginea* extracts; scavenging activity increased significantly with increasing concentration. The antioxidant activity of *S*. *ferruginea* extracts varied depending on the solvent used for extraction. This study showed that change in solvent polarity alters its extraction efficacy for particular group of antioxidants and influences the scavenging capacity of the extracts. For example, the methanolic extracts exhibited better antioxidant values compared to the aqueous extracts. IC_50_ is the main parameter to determine the antioxidant activity. A lower IC_50_ indicates a higher scavenging activity. IC_50_ was on the lower side when using methanolic extracts (stem: 27.81 (95% CI: 24.71–31.29) μg/mL, leaf: 40.29 (95% CI: 35.75–45.41) μg/mL, and flower: 33.35 (95% CI: 29.40–37.84) μg/mL). It showed that these extracts have significant radical scavenging activity resulting from the reduction of DPPH^•^. The stem methanol extract showed the highest antioxidant activity (96.4%) when used at the highest tested concentration. The higher DPPH^•^ scavenging ability of the methanol extracts may be attributed to the presence of a higher content of polyphenols and flavonoids. On the other hand, higher IC_50_ values were obtained for *S*. *ferruginea* hexane extracts (stem: 194.4, leaf: 235.4, and flower: 217.8) compared to other solvent extracts. It indicated that these extracts possess low ability for scavenging free radicals.

### Fe^2+^-chelating ability

**Fig 2:**
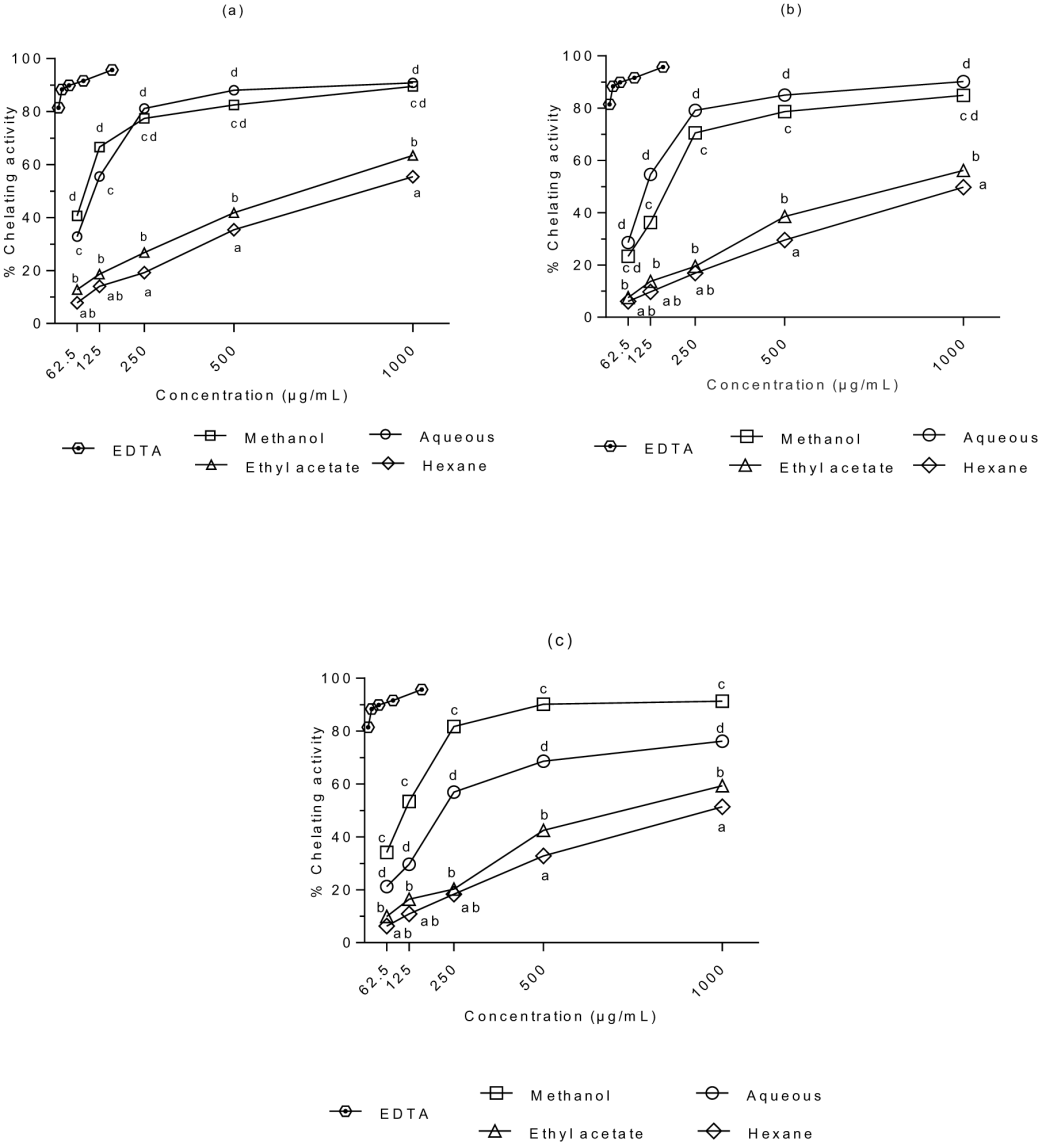
Metal chelating activities of (a) stem, (b) leaf, and (c) flower extracts of *S*. *ferruginea* and positive control (EDTA). Results are means ± SD (n = 3). Points marked with different letters are significantly different at *P* < 0.05 when compared at the same concentration determined by two-way ANOVA. The positive control (EDTA) showed a significantly higher scavenging capacity as compared to the samples (*P* < 0.05).

The abilities of *S*. *ferruginea* extracts to chelate Fe^2+^ are presented in Fig 2. Three-way ANOVA demonstrated that S, P, C, and S × P × C exhibited significant effects on metal chelation activity of the extracts (p < 0.05). However, P × C exhibited no significant effect on metal chelation activity (P > 0.05). Furthermore, there were significant differences between the stem and leaf extracts, and between the stem and flower extracts (*P <*0.05). However, there was no significant difference in the Fe^2+^ chelating capacity of leaf and flower extracts (P > 0.05). All samples showed significantly lower Fe^2+^ chelating effects (P < 0.05) compared to EDTA (control). The chelating effects of the extracts increased significantly with increasing concentration. The methanol and aqueous extracts exhibited higher chelating activity than the ethyl acetate and hexane extracts at tested concentrations. Similar to the DPPH^•^ scavenging activity, the Fe^2+^ chelating ability showed dose dependent increase of decrease; higher dose produced higher metal chelation activity. The leaf hexane extract presented the lowest Fe^2+^-chelating capacity. Based on the ability to chelate Fe^2+^, it can be suggested that the methanolic extract of *S*. *ferruginea* may be a good source of antioxidant polyphenols.

### Determination of TEAC value

The TEAC values for the investigated extracts are shown in Table 4. The ABTS^•+^ scavenging capacity ranged from 353.9 ± 4.40 to 1483.4 ± 4.05 μM Trolox/g extract. Two-factor ANOVA revealed a significant influence of P, S, and S × P (P < 0.05) on the ABTS^•+^ scavenging ability. The radical scavenging capacities of flower, stem, and leaf extracts were significantly different (*P <* 0.05). Although the stem aqueous extract showed the highest ABTS^•+^ scavenging ability, there was no significant difference between the stem aqueous and methanolic extracts (P > 0.05). Two-way ANOVA showed a significant difference (p<0.05) between the solvents groups; however, there was no significant difference between the methanolic flower and stem extracts (P > 0.05). Similar to the DPPH^•^ scavenging activity, plant part and solvent polarities affected the ABTS^•+^ scavenging capacity.

**Table 4:** ABTS^•+^ radical scavenging capacity of *S*. *ferruginea* extracts. Data are expressed as μM Trolox/g extract.

		TEAC Value ^a^		
		Extractants		
Plant part	Water	Methanol	Ethyl acetate	Hexane
**Stem**	1483.4 ± 4.05^ax^	1477.2 ± 4.39^ax^	1140.7 ± 7.33^bx^	688.6 ± 4.05^cx^
**Leaf**	1220.9 ± 8.16^ay^	1435.0 ± 1.99^by^	1012.7 ± 7.77^cy^	456.7 ± 7.79^dy^
**Flower**	1322.7 ± 3.78^az^	1470.5 ± 4.49^bx^	833.8 ± 9.71^cz^	353.9 ± 4.40^dz^

All analysis were performed in triplicate and expressed as means ± SD. For the same extract with different part of plant, means in the same column with different letters (x-z) are significantly different (p < 0.05, two-way ANOVA). For different extracts with the same part of plant, means in the same row with different letters (a-d) show significant difference (p < 0.05, two-way ANOVA).

### Correlation between antioxidant activity assays, phenolic and flavonoid contents

Table 5 presents the correlation coefficients of the possible correlation between the phenolic and flavonoid contents of *S*. *ferruginea* extracts and their antioxidant activities. It also presents the correlation between different methods used. The TPC and TFC exhibited a significant and positive linear correlation (p<0.05) with different antioxidant activity assays. The correlation had a decreasing order of TPC>TFC. These results suggested that the antioxidant activity is more closely related to TPC than TFC. A higher positive correlation between TPC and antioxidant activity assays also proved that the phenolic compounds were the major contributors to the antioxidant capacity of the *S*. *ferruginea* extracts. Furthermore, Pearson correlation analysis of the results revealed a significant and positive correlation between different antioxidant assays (p<0.05). The highest correlation was found between ABTS and DPPH (r = 0.933), whereas ABTS and metal chelation activity showed the lowest correlation (r = 0.855). It indicated that the compounds in the extracts that could scavenge ABTS^•+^ were also able to scavenge DPPH^•^ and act as metal chelators. Furthermore, the strong correlation between DPPH and ABTS methods suggests that the antioxidants in the extracts react similarly with both assays.

**Table 5:** Pearson’s correlation coefficients of antioxidant activities, TFC, and TPC of *S*. *ferruginea* extracts.

Trait ^a^	TPC	TFC	DSC	MCA	ASA
**TPC**	1	0.942**	0.809**	0.861**	0.817**
**TFC**		1	0.800**	0.855**	0.816**
**DSC**			1	0.862**	0.933**
**MCA**				1	0.855**
**ASA**					1

^a^ TPC; total phenolic content, TFC; total flavonoid content, DSC; DPPH^•^ scavenging capacity, MCA; metal chelation activity, ASA; ABTS^•+^ scavenging activity.

**significant at P < 0.05.

### *In vitro* cytotoxic activity of methanolic and aqueous extracts of *S*. *ferruginea* stem

**Fig 3:**
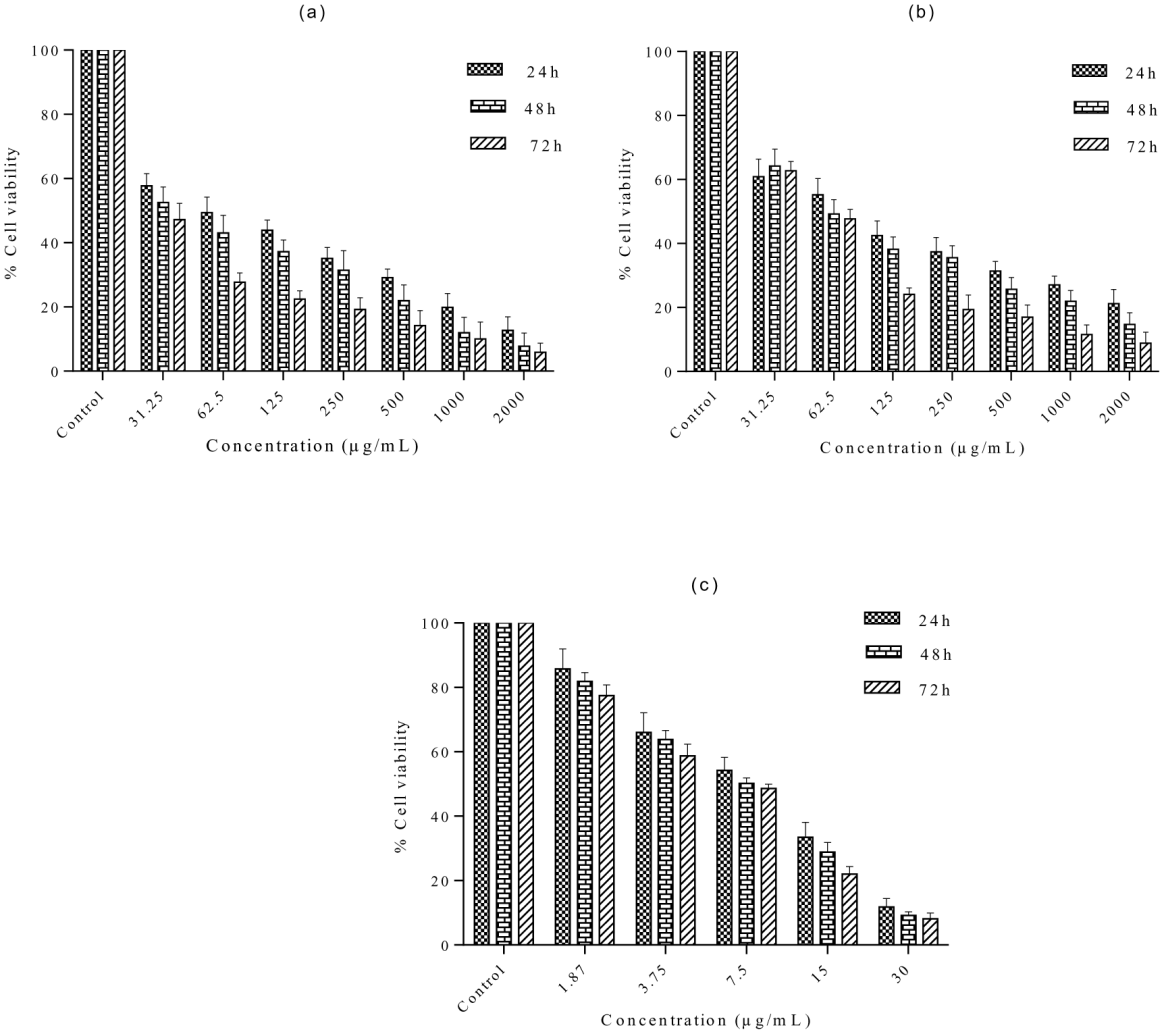
*S*. *ferruginea* extracts inhibit MDA-MB-231 proliferation in a time- and dose-dependent manner. The cells were treated with indicated concentrations of (a) methanol and (b) aqueous extracts of *S*. *ferruginea* for indicated time intervals. The results are expressed as the percentage of the negative control. Cytotoxic activity of the extracts was compared to the reference drug, tamoxifen (Fig 3C). Cell viability was measured by MTT assay. Data are means ± SD of triplicate in three independent experiments. All data showed statistically significant difference from untreated control (one-way ANOVA, P < 0.05).

Because the stem aqueous and methanolic extracts exhibited strong antioxidant potential, these extracts were used for further cytotoxicity studies. The antiproliferative effects of the methanolic and aqueous extracts of *S*. *ferruginea* stems against MDA-MB-231 cells are shown in Fig 3. To our knowledge, this is the first study that evaluated the cytotoxic potential of *S*. *ferruginea* stem extracts against a breast cancer cell line. A significant difference was found between extract-treated cells and control (0.1% DMSO) as well as positive control (tamoxifen). The results indicated that methanolic and aqueous extracts reduced the viability of MDA-MB-231 cells in a time- and dose-dependent manner. The IC_50_ for *S*. *ferruginea* extracts and tamoxifen is shown in Table 6. Tamoxifen was more potent cytotoxic than the tested extracts of the plant. IC_50_ of the aqueous and methanolic extracts in MDA-MB-231 cells was 50.35 (95% CI: 41.26–61.44) μg/mL and 19.27 (95% CI: 13.11–28.32) μg/mL, after 72 h treatment, respectively. Pair-wise comparison between the IC_50_ values of the methanolic and aqueous extract showed that the latter was less effective in suppressing the growth of breast cancer cell line. In addition, the maximum antiproliferative activity (nearly 95% reduction in cell viability) was observed after 72 h treatment with methanolic extract at the highest dose (2 mg/mL). Similarly, a 72 h treatment with the same dose (2 mg/mL) of the aqueous extract produced 91% reduction in cell viability. However, *S*. *ferruginea* extracts showed higher cytotoxic activity than negative control after 72 h incubation.

**Table 6:** Inhibitory effect of *S*. *ferruginea* extracts against MDA-MB 231 breast cancer cell line at different incubation times.

		IC_50_ (μg/mL)^a^	
Extracts	24 h	48 h	72 h
**Methanol**	65.19	41.99	19.27
**95% CI**	53.22–79.86	31.42–56.11	13.11–28.32
**Aqueous**	82.60	69.63	50.35
**95% CI**	65.36–104.4	56.16–86.32	41.26–61.44
**Tamoxifen**^b^	7.71	6.66	5.64
**95% CI**	6.73–8.82	5.92–7.49	5.00–6.35

^a^ Data are expressed as IC_50_ and the values are presented with their respective 95% confidence interval (95% CI).

^b^ Positive reference standard.

**Fig 4:**
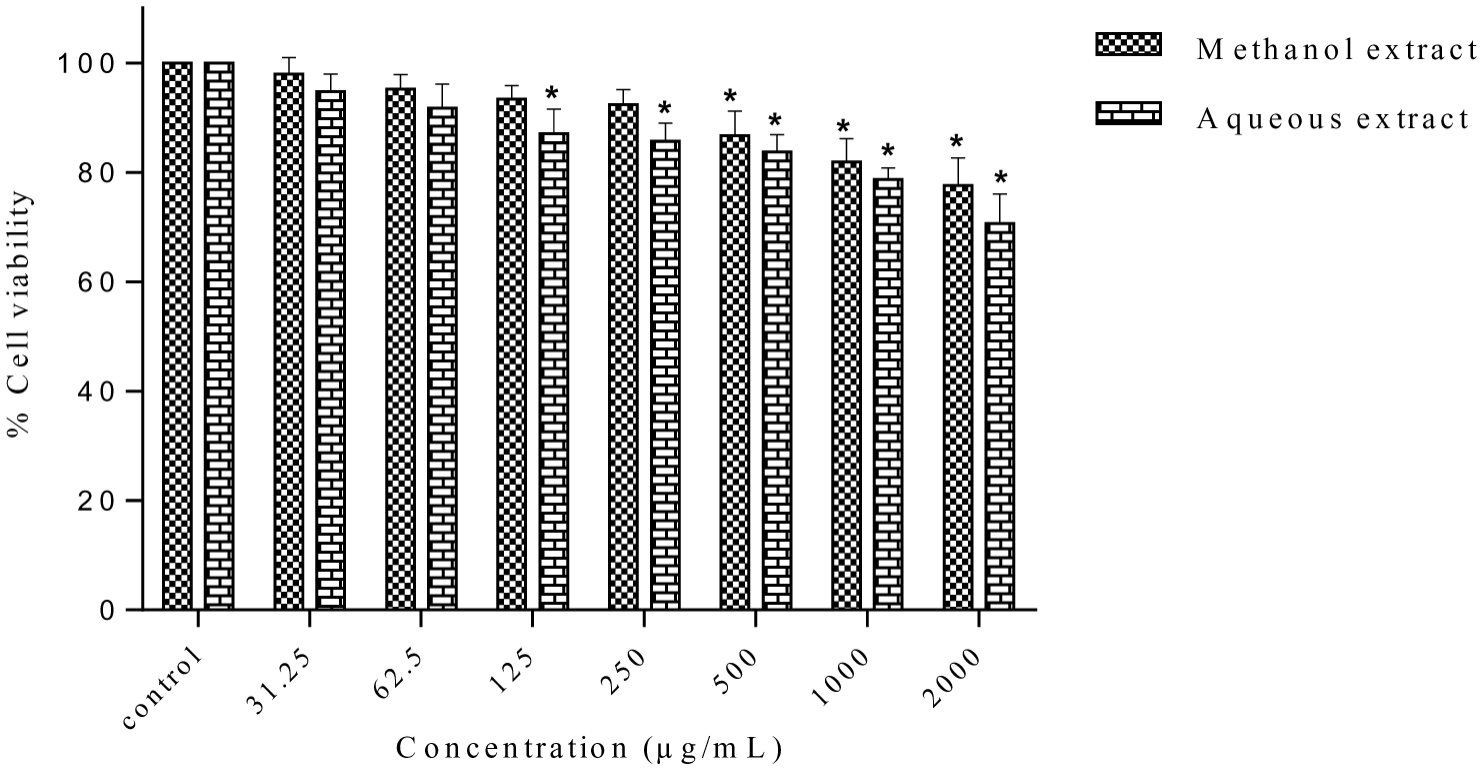
*In vitro* antiproliferative activity of the methanolic and aqueous extracts of *S*. *ferruginea* stems against HSF-1184 normal cell line. The results are expressed as the percentage of the control. The extracts were non-selective and showed negligible toxicity in normal cell line. Data are presented as means ± SD of triplicate in three independent experiments. * indicates statistically significant difference compared to untreated control (one-way ANOVA, P < 0.05).

In order to investigate the cytotoxic effect of *S*. *ferruginea* extracts towards normal cells, HSF-1184 cells were incubated with different concentrations of the methanolic and aqueous extracts for 24, 48, and 72 h. As shown in Fig 4, both the methanolic and aqueous extracts of *S*. *ferruginea* did not affect the viability of normal cells (HSF-1184). The IC_50_ obtained for normal cells was higher than that in cancer cells (Table 7). Collectively, these results suggest that *S*. *ferruginea* extracts are selective for breast cancer cells but non-toxic to normal cells.

**Table 7:** Effect of *S*. *ferruginea* extracts on HSF-1184 expressed as IC_50_ values at different incubation time.

		IC_50_ (μg/mL)^a^	
Extract	24 h	48 h	72 h
**Methanol**	ND	>500	474.4 (435.2–517.2)^a^
**Aqueous**	ND	>500	>500

ND: not detected within the investigated concentration range.

^a^ 95% confidence interval.

### Morphological changes of MDA-MB-231 cells following treatment with *S*. *ferruginea* extracts

**Fig 5:**
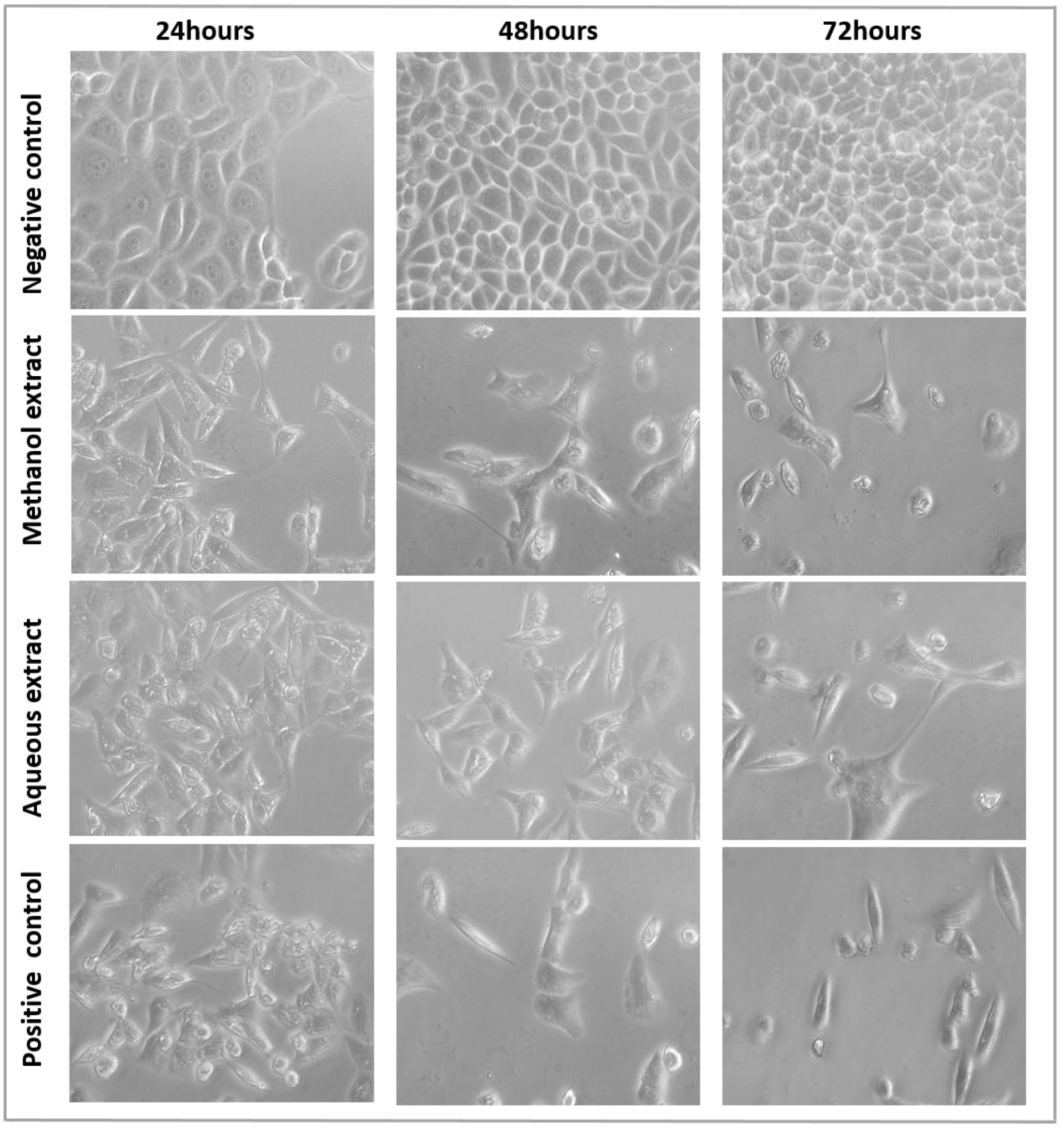
The morphological changes of MDA-MB-231 cells treated with methanolic and aqueous extracts at their respective IC_50_. MDA-MB-231 cells treated with 8.5 μg/mL tamoxifen were used a positive control. After 24, 48, and 72 h of treatment, the cell morphological alterations were observed with an inverted-phase contrast microscope.

The methanol and aqueous extracts showed antiproliferative activity in breast cancer cells. Therefore, it was investigated whether the cytotoxic effects of *S*. *ferruginea* extracts were linked to its ability to induce apoptosis in MDA-MB-231 cells. As shown in Fig 5, the untreated MDA-MB-231 cells exhibited normal morphology (well spread and flattened) and normal rate of proliferation. In contrast, exposure to extracts produced typical apoptotic features. Morphological alterations were concentration and time-dependent (only images corresponding to IC_50_ are shown in Fig 5). A clear reduction of cellular and nuclear volume, increased number of floating dead or dying cells, cytoplasmic condensation, and cell shrinkage, (which is suggestive of cell death) were observed at 48 and 72 h after treatment with 125, 250, and 500 μg/mL of extracts (images not shown). When treated with 1000 μg/mL of the methanolic and aqueous extracts, most of the cells detached from the surface of the tissue culture plates and floated in the culture medium. These cells lost their typical morphology, and appeared smaller and rounded.

### Apoptosis detection by acridine orange/ethidium bromide staining

**Fig 6:**
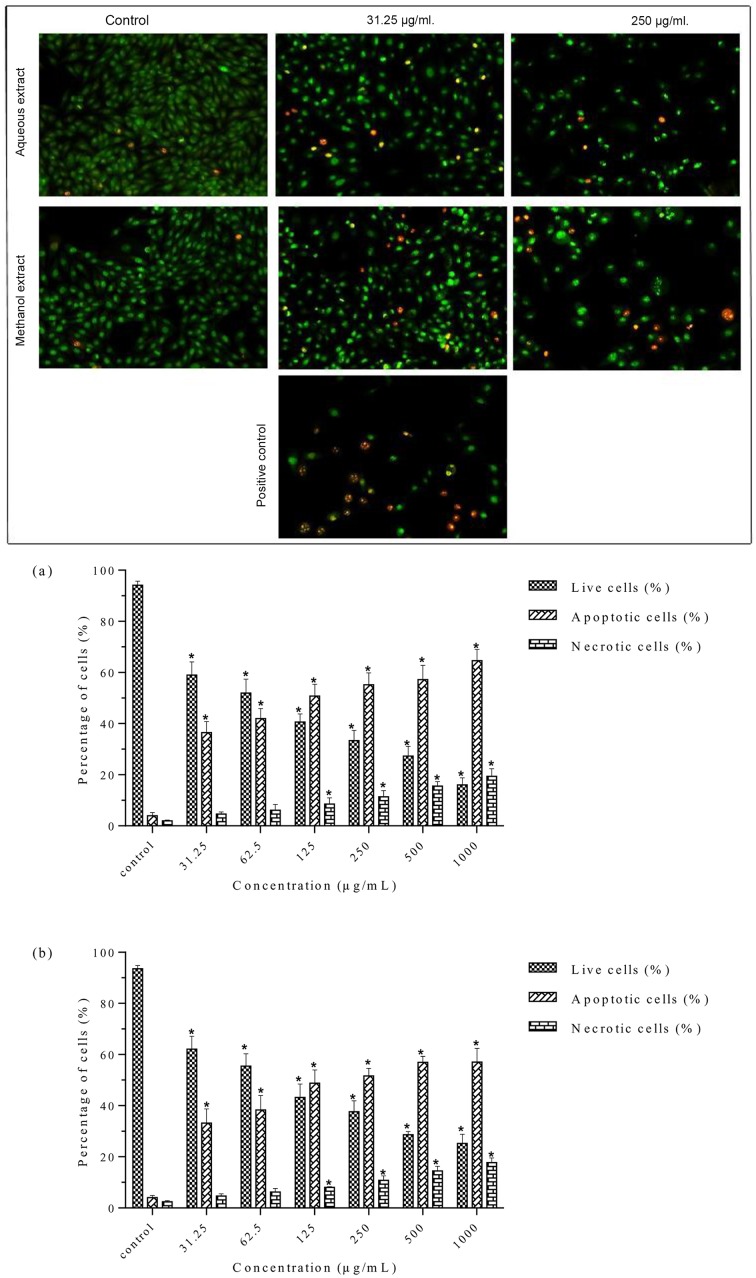
Morphological observation of AO/EB-stained MDA-MB-231 cells. A. Cells incubated for 24 h with methanolic (left panel) and aqueous (right panel) extracts. Cells treated with 8.5 μg/mL tamoxifen were used as a positive control. The morphological alterations in the cells were visualized under a fluorescence microscope (magnification: 200x). Viable cells stained uniformly in green color and showed normal morphology. Treated cells showed early apoptotic cells with membrane blebbing and bright green nuclei, late apoptotic cells with fragmented and condensed orange-red nuclei, and necrotic cells with deep orange nucleus. B. The percentage of live, apoptotic, and necrotic cells at different concentration (31.25–1000 μg/mL) of (a) methanolic and (b) aqueous extracts. Data are means ± SD of triplicate in three independent experiments. A minimum of 200 cells were counted for each replicate. * indicates statistically significant difference from their respective untreated control (one-way ANOVA, P < 0.05).

The microscopic examination showed that the live cells had a normal morphology, took up AO, and stained uniformly in green color. In contrast, cells treated with the methanolic and aqueous extracts showed characteristics of cell death and altered cell morphology such as condensed and fragmented chromatin, cellular shrinkage, and membrane blebbing. Early apoptotic cells with bright green nuclei and late apoptotic cells with condensed orange-red nuclei were observed in extract-treated cells (Fig 6A). There was a significant increase in apoptotic cells and apoptotic bodies after treatment with both methanolic and aqueous extracts compared to the control. This increase was dose dependent as shown in the histogram (Fig 6B). The percentage of apoptotic cells with membrane blebbing and orange condensed chromatin was 36.44 ± 4.35% and 33.22 ± 5.49% at 31.25 μg/mL for the methanolic and aqueous extracts, respectively. The percentage of apoptotic cells (apoptotic index) with more condensed and fragmented chromatin increased sharply at higher concentrations. Furthermore, the percentage of necrotic cells with deep-orange nuclei increased sharply at 500 μg/mL and 1000 μg/mL for both the methanolic and aqueous extracts. It may correspond partly to a final phase of apoptosis, when the cytoplasmic membrane integrity is lost. The methanolic extract induced higher percentage of apoptotic cells than the aqueous extract. Taken together, these results indicated that *S*. *ferruginea* methanolic and aqueous extracts show cytotoxicity against breast cancer cells by inducing cell death through an apoptotic pathway.

### Nuclear morphological studies using PI and Hoechst staining

**Fig 7:**
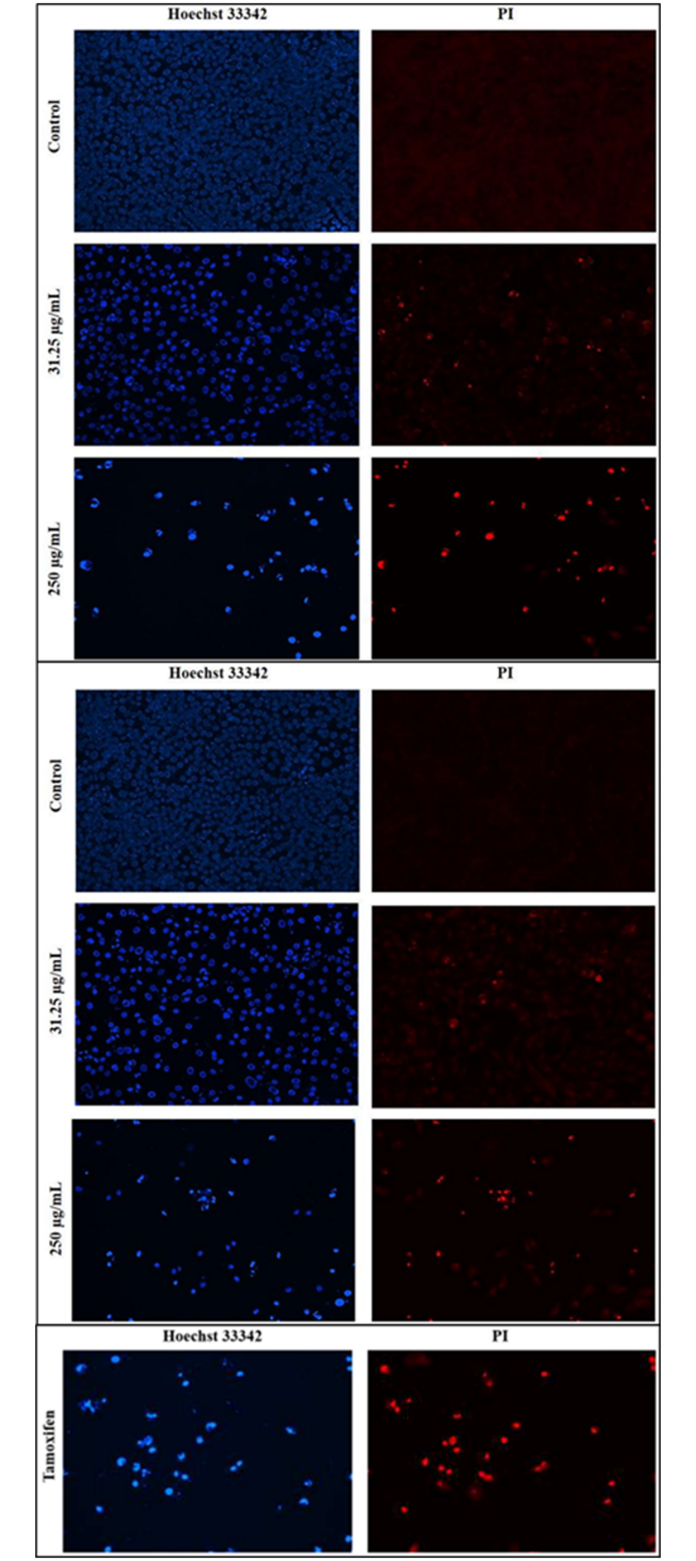
Fluorescence imaging for detection of apoptosis in MDA-MB-231 cells. Cells were treated with A) methanolic extract B) aqueous extract, and C) positive control (tamoxifen) for 24 h at 31.25 and 250 μg/mL. Left panel displays Hoechst 33342 staining while right panel displays PI staining of the same field. The morphological alterations in cells were visualized under a fluorescence microscope (magnification: 200x). Extract-treated cells showed condensed and fragmented nuclei at both tested concentrations. The number of distorted nuclei and apoptotic cells were higher at 250 μg/mL than at 31.25 μg/mL. The percentage of apoptotic cells was higher among cells treated with the methanolic extract than among those treated with the aqueous extract.

The cell nuclei were stained with DNA-specific fluorochromes, PI and Hoechst 33342, to verify AO/EB staining results. Control cells showed regular intact nuclei with minimal debris. The nuclei of control cells showed a less bright blue fluorescence after staining with Hoechst 33342 and an absence of red fluorescence after staining with PI (as it is effluxed out by living cells). On the other hand, nuclei of extract-treated cells stained with Hoechst 33342were smaller and showed a brighter blue fluorescence than the control cells. Furthermore, extract-treated cells were stained red by PI, which can stain all of the dead cells including late apoptotic and necrotic. The nuclear morphology of cells treated with *S*. *ferruginea* extracts showed distinguishable changes (Fig 7). Extract-treated cells showed apoptotic features like condensed nuclei and formation of apoptotic bodies (only images corresponding to 31.25 and 250 μg/mL are shown in Fig 7). The number of distorted nuclei and apoptotic cells increased when the cells were treated with higher concentration (250 μg/mL) of extracts. Furthermore, the frequency of the deformed nuclei was lower in cells treated with the aqueous extract than in the methanolic extract.

### Effect of *S*. *ferruginea* extract on colony formation in MDA-MB-231 cells

**Fig 8:**
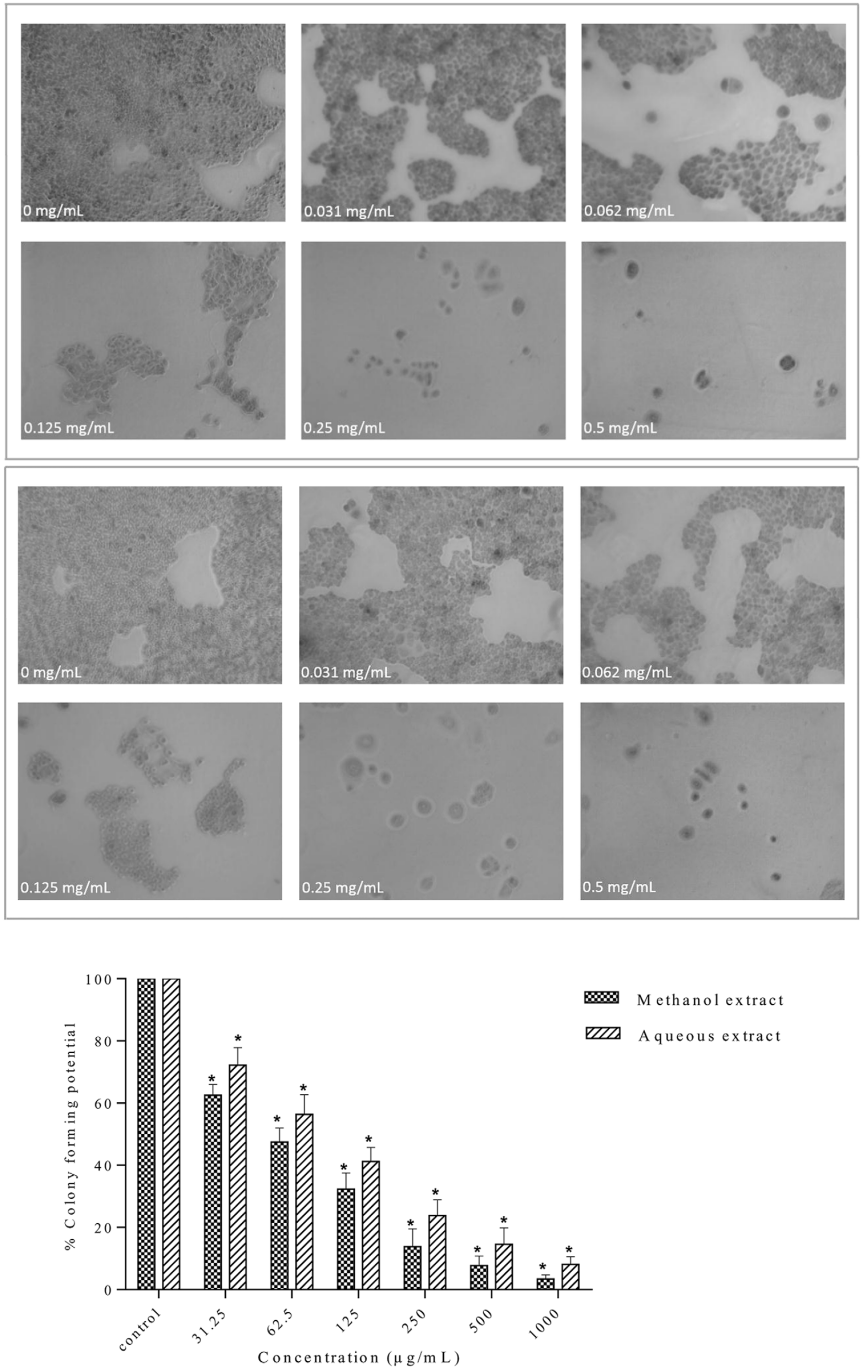
Effect of *S*. *ferruginea* extracts on colony-forming abilities of MDA-MB-231 cells. (A). Methanolic (a) and aqueous (b) extracts suppressed colony formation in a dose dependent manner. The methanolic extract inhibited the clonogenicity of MDA-MB-231 cells more effectively than the aqueous extract. (B) Quantitative measurement of colony formation of selected extracts on MDA-MB-231 cells at different concentration (31.25–1000 μg/mL). The colony forming ability of the cells at each dose of the extract is expressed in terms of percent of untreated control cells. Data are means ± SD of triplicate in three independent experiments. * indicates statistically significant difference from their respective untreated control (one-way ANOVA, P < 0.05).

Both methanolic and aqueous extracts showed growth inhibitory potential against MDA-MB-231 cells as described earlier. Clonogenic inhibition assay was used to examine and verify the cytotoxic effects of *S*. *ferruginea* extracts. Similar to the cytotoxic activities, the colony formation was proportional to the extract concentrations. As shown in Fig 8, both the methanolic and aqueous extracts inhibited the colony formation ability of MDA-MB-231 cells in a concentration-related manner. The methanolic and aqueous extracts at different concentrations, particularly at 1000 and 500 μg/mL, markedly reduced the size and the number of colonies. The methanolic extract inhibited the colony-forming abilities of cells better than the aqueous extract. Even at the lowest tested concentration (31.25 μg/mL), there was a significant difference in colony forming potential exerted by the methanolic and aqueous extracts compared to negative control (p<0.05). These results demonstrated that *S*. *ferruginea* extracts, particularly the methanolic extract, had a cytostatic effect on long-term colony formation in MDA-MB-231 cells.

### Cell migration-inhibition efficiency of *S*. *ferruginea* extracts

**Fig 9:**
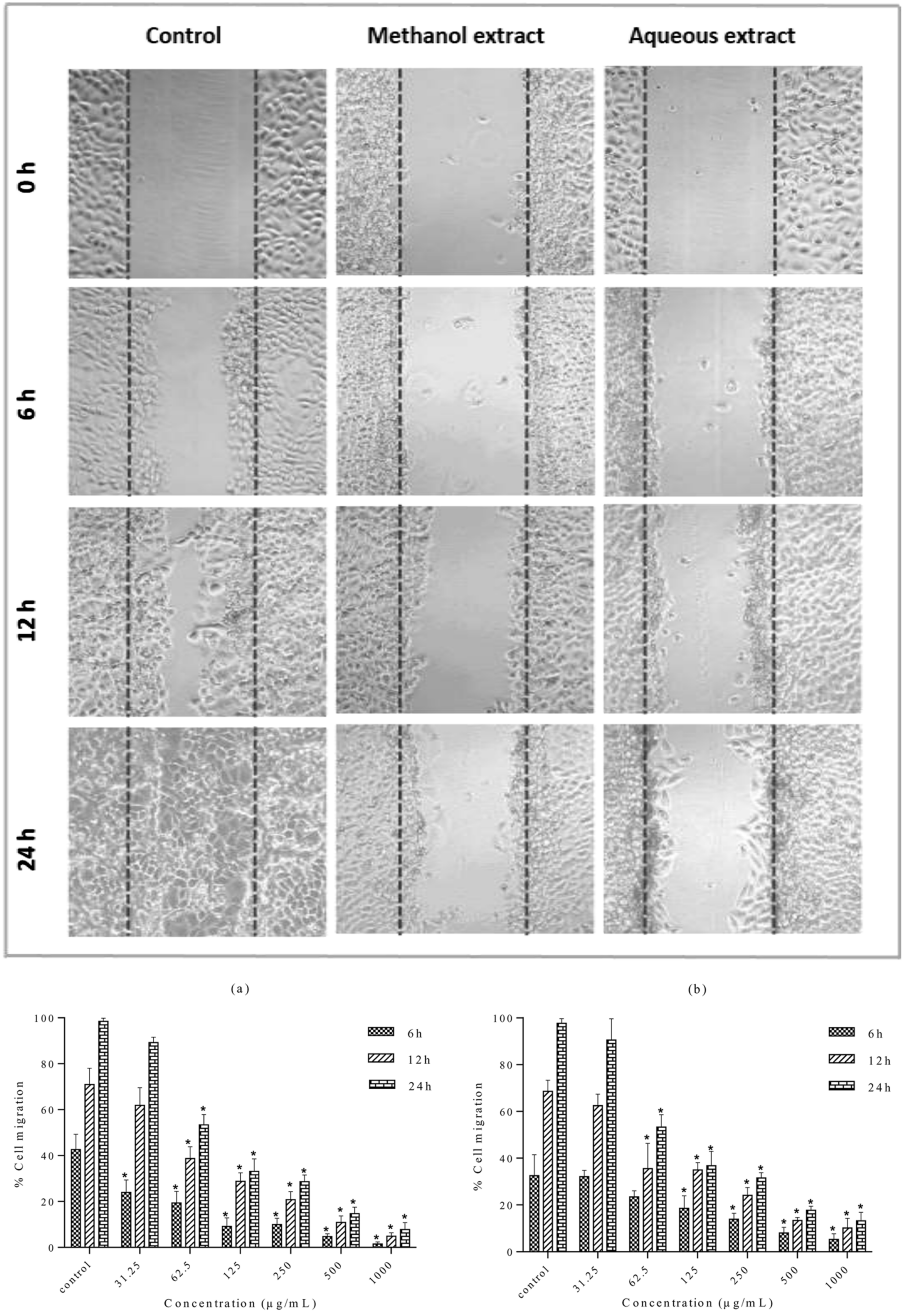
Effect of *S*. *ferruginea* methanolic and aqueous extracts on the cell migration of MDA-MB-231 cells. (A) Wound closure ability of treated MDA-MB-231 cells after creation of scratch wound in control and treated well. The images of wounded extract-treated MDA-MB-231 cell monolayers captured using an inverted phase-contrast microscope at different time intervals (0, 6, 12, and 24 h) are shown. (B) Quantitative measurement of MDA-MB-231 cell migration after treatment with methanolic (a) and aqueous (b) extracts at different concentrations (31.25–1000 μg/mL). Wound closure rates were quantitatively analyzed by calculating the difference between wound width at 0, 6, and 12 or 24 h of extract-treated and control cells. Results are expressed as percentage of cell migration. Data are means ± SD of triplicate in three independent experiments. * indicates statistically significant difference from their respective untreated control (one-way ANOVA, P < 0.05).

The cells in the control wells migrated along the edges of wound and covered (healed) the wound attaining a complete confluency after 24 h. In contrast, cell migration was significantly inhibited after treatment with the methanolic and aqueous extracts (Fig 9A). MDA-MB-231 cell migration inhibition by the methanolic and aqueous extracts was quantitatively analyzed at different concentrations (31.25–1000 μg/mL). Results were expressed as percentage of cell migration (Fig 9B). Treatment with the methanolic and aqueous extracts inhibited the migration of MDA-MB-231 cells dose- and time-dependently. The methanolic extract inhibited the migration more effectively than the aqueous extract. The methanolic and aqueous extracts exhibited significantly lower percentage cell migration rates than negative control (p<0.05) after 24 h treatment at 62.5–1000 μg/mL. However, there was no significant difference in migratory capacity when cells were treated with lowest concentration (31.25 μg/mL) of both extracts compared to negative control (p>0.05). It can be suggested that the methanolic and aqueous extracts exerted antimetastatic activity due to the antiproliferative and cytotoxic activity at high concentrations.

### Measurement of ROS generation in MDA-MB-231 cells

**Fig 10:**
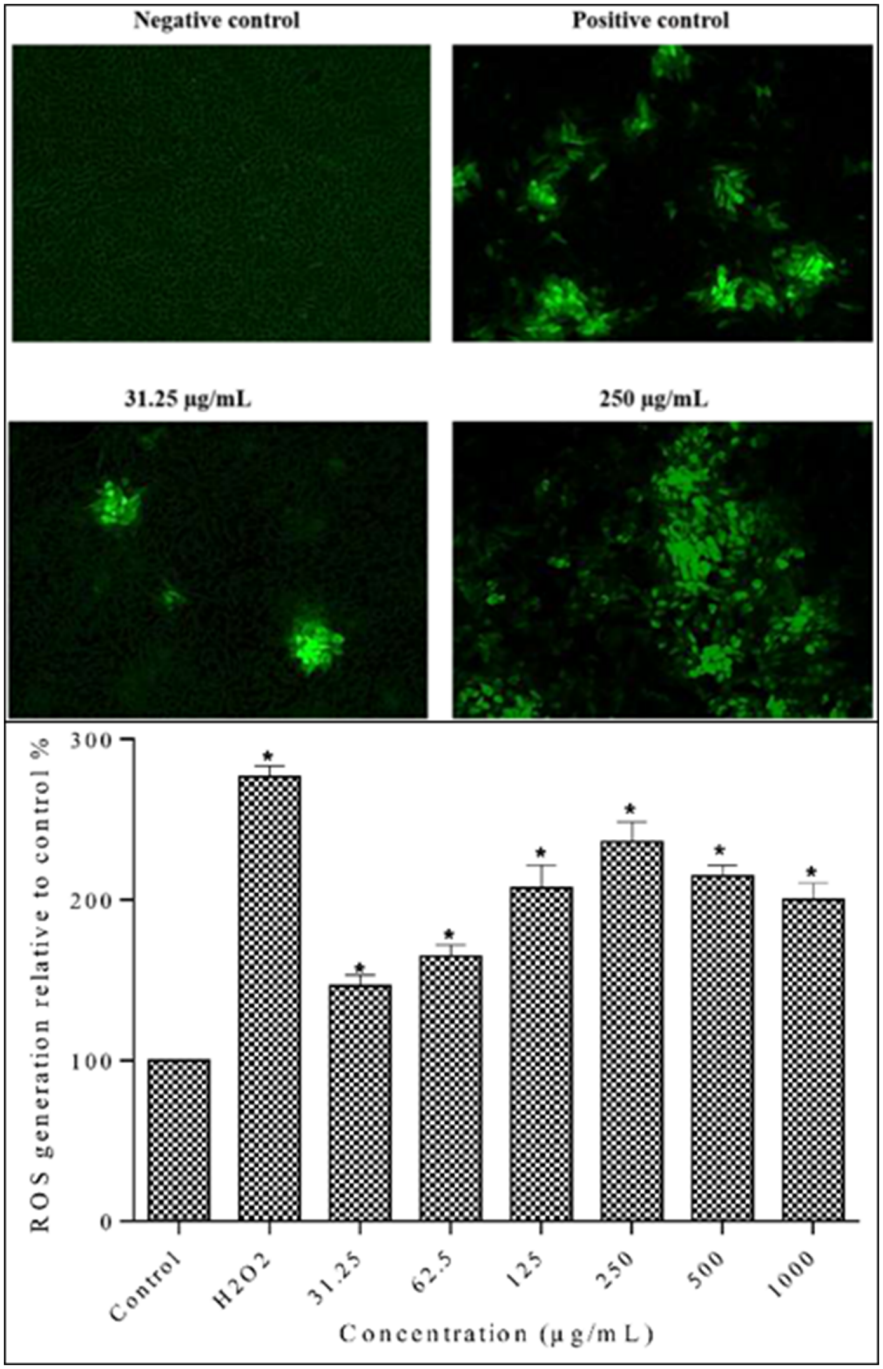
Evaluation of ROS generation in MDA-MB-231 cells using the fluorescent probe DCF-DA. A. Qualitative measurement of intracellular ROS levels in MDA-MB-231 cells fluorescence images were taken at 10x magnification. Cells were treated with extracts at 31.25 μg/mL and 250 μg/mL and with positive control (50 μM H_2_O_2_) for 24 h. Fluorescence images indicated that methanolic extract induced intracellular ROS generation in MDA-MB-231 cells. B. Quantitative measurement of intracellular ROS levels in MDA-MB-231 cells. Cells were treated with different concentrations of methanolic extract and positive control (50 μM H_2_O_2_) for 12 h. Data are means ± SD of triplicate in three independent experiments. * indicates statistically significant difference from corresponding controls (one-way ANOVA, P < 0.05).

Since ROS generation is an early event in apoptosis, the ROS production in cells was determined after 12 h [42]. As shown in Fig 10A, control group showed faintly green fluorescence, indicating that ROS formation was at basal level. The lowest concentration (31.25 μg/mL) of the methanolic extract had the lowest effect on ROS generation. On the other hand, positive control and 250 μg/mL incubation groups induced bright green fluorescence showing a large amount of ROS generation.

The methanolic extract increased ROS generation in breast cancer cells in a concentration dependent manner (Fig 10B). The ROS generation was significantly increased particularly at higher concentrations (250, 500 μg/mL). The highest ROS generation in MDA-MB-231 cells was observed at 250 μg/mL. However, ROS generation decreased at highest concentration (1000 μg/mL), which might be due to the apoptotic and necrotic cell death at high concentration. Taken together, results demonstrated that the *S*. *ferruginea* stem methanolic extract induced mitochondrial mediated apoptosis in MDA-MB-231 cells due to ROS hyper-generation.

### Measurement of mitochondrial membrane potential (MMP) by JC-1 assay

**Fig 11:**
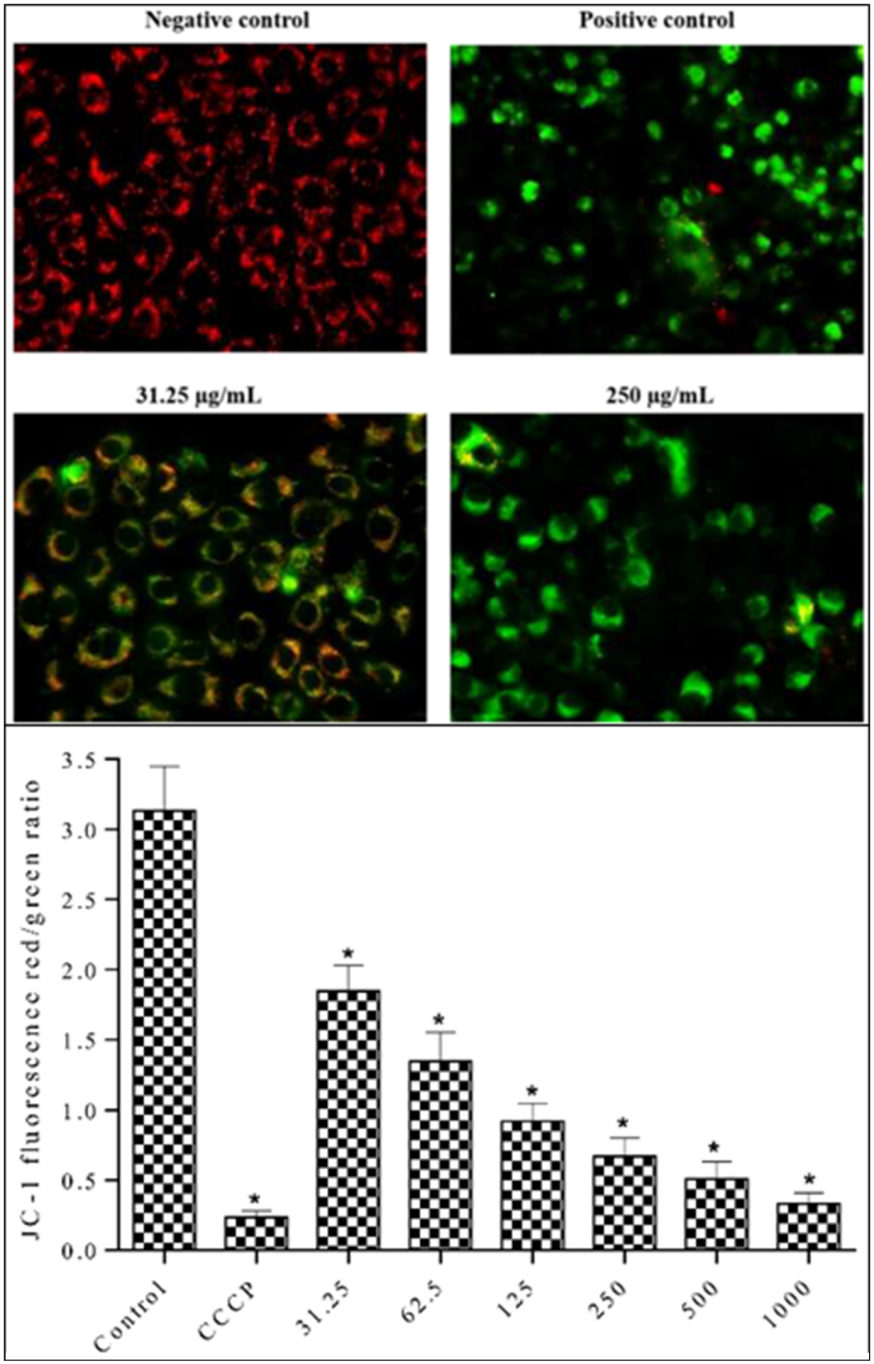
Effect of methanolic extract on MMP in MDA-MB-231 cells using JC-1 fluorescence dye. A. Methanolic extract induced MMP depolarization in MDA-MB-231 cells. The cells were treated with extracts at 31.25 μg/mL and 250 μg/mL and with positive control (50 μM CCCP) for 12 h. Cells were imaged with an inverted fluorescence microscope (Zeiss Axiovert A) at 40x magnification. The emitted green fluorescence indicates MMP depolarization, which is an early event in apoptosis. B. Relative quantification of MMP (*ΔΨ*m) in MDA-MB-231 cells. Cells were treated with different concentrations of methanolic extract and positive control (50 μM CCCP) for 12 h. Methanolic extract disrupts MMP (*ΔΨ*m). Data are means ± SD of triplicate in three independent experiments. * indicates statistically significant difference from corresponding controls (one-way ANOVA, P < 0.05).

Since high ROS generation could lead to mitochondrial damage, MMP was determined using the membrane-permeant JC-1 dye. MDA-MB-231 cells were treated with the methanolic extract for 24 h to determine the MMP changes. Carbonyl cyanide m-chlorophenyl hydrazone (CCCP) served as positive control (50 μM). MMP is lost in apoptotic cells. Apoptotic cells stained with JC-1 form a monomer that shows green fluorescence. In contrast, JC-1 forms aggregates with red fluorescence in cells with healthy mitochondria. As shown in Fig 11A, control group treated with 0.1% DMSO, showed orange-red fluorescence due to high MMP (ΔΨm). The cells treated with the lowest concentration of the methanolic extract (31.25 μg/mL) showed both red orange and green fluorescence. The cells treated with 250 μg/mL methanolic extract showed progressive loss of red JC-1 aggregate fluorescence. Furthermore, the green monomer fluorescence diffused to cytoplasm indicating depolarization of MMP. This can be a possible mechanism for the extract-induced apoptotic pathway. Loss of red fluorescence and retention of green fluorescence was highest in positive control group, indicating its potent apoptotic activity. The MMP, which is an indicator of mitochondrial-dependent apoptosis, was quantitatively measured by spectrofluorometric analysis using JC-1 dye. The alterations in the ratio of red to green fluorescence intensities are indicative of changes in the *ΔΨ*m [43, 44]. A 12 h exposure of MDA-MB-231 cells to *S*. *ferruginea* extract decreased the ratio of red-green fluorescence intensity in a concentration-dependent manner, indicating disruption of MMP (Fig 11B). Treatment of cells with all extract concentrations produced significant depolarization of mitochondrial membranes compared to the control (0.1% DMSO).

### Effect of methanolic extract on pro-apoptotic and anti-apoptotic proteins

**Fig 12:**
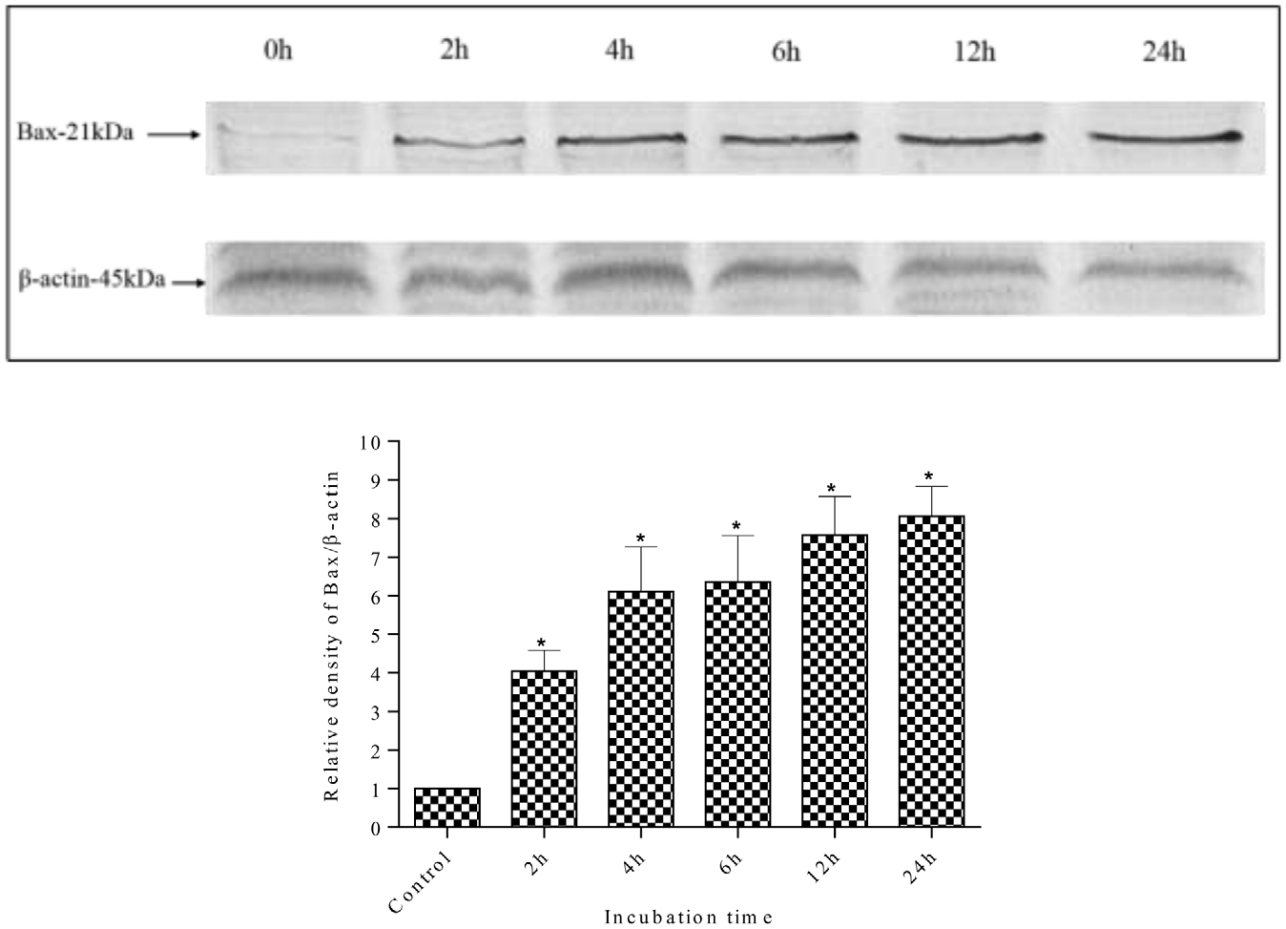
Western blot analysis of pro-apoptotic Bax protein in MDA-MB-231 cells. MDA-MB-231 cells were treated with *S*. *ferruginea* methanol extract at IC_50_ for indicated times. Control cells were treated with 0.1% DMSO. β-Actin was used as loading control. Densitometric analysis showed time-dependent up-regulation of Bax protein. The expression of Bax protein increased as early as after 2 h extract-treatment. The densitometric-intensity data are presented as means ± SEM of triplicate in three independent experiments. * indicates statistically significant difference from control (one-way ANOVA, P < 0.05).

**Fig 13:**
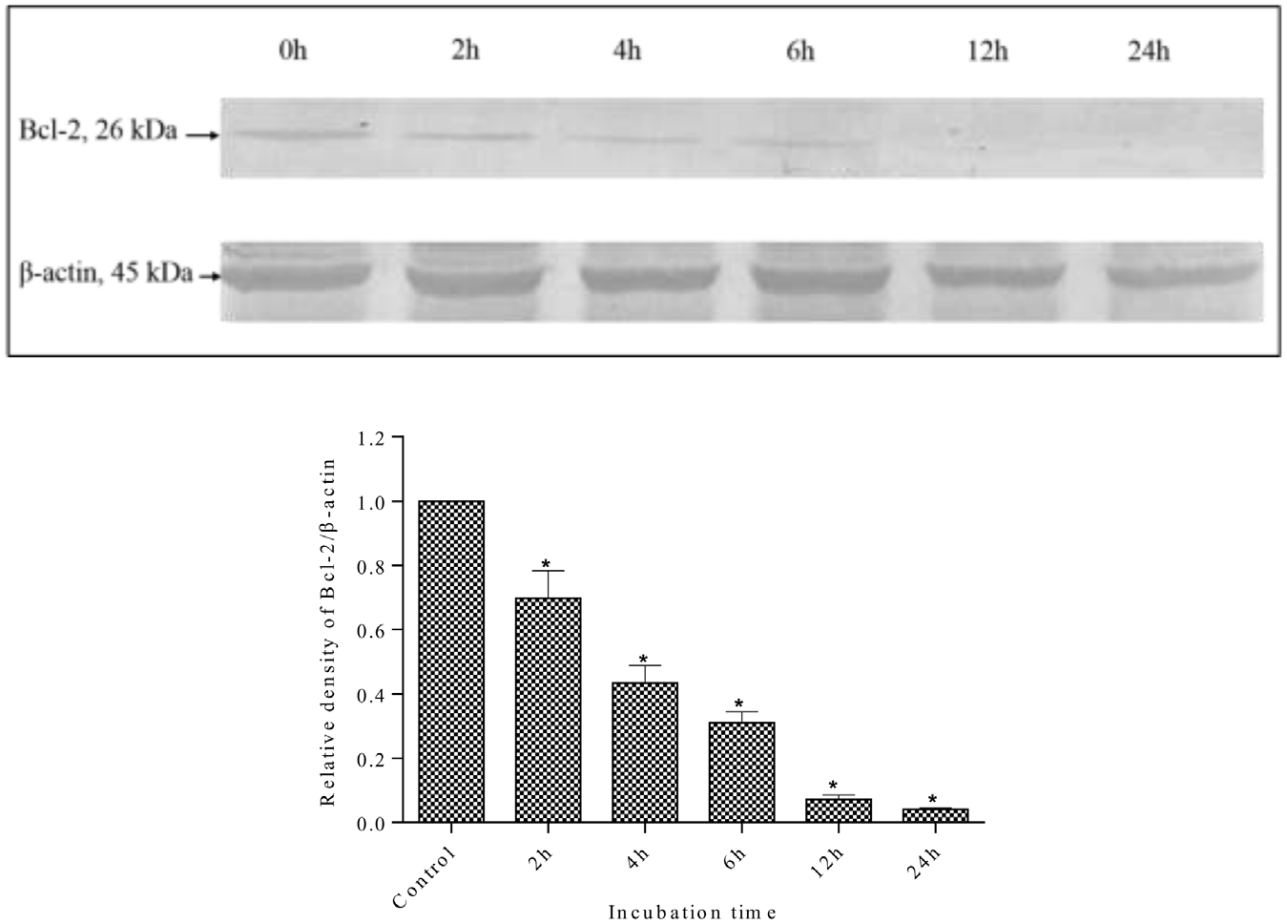
Western blot analysis of anti-apoptotic Bcl-2 protein in MDA-MB-231 cells. MDA-MB-231 cells were treated with *S*. *ferruginea* methanol extract at IC_50_ for indicated times. Control cells were treated with 0.1% DMSO. β-Actin was used as loading control. Densitometric analysis showed time-dependent down-regulation of Bcl-2 protein. The densitometric-intensity data are presented as means ± SEM of triplicate in three independent experiments. * indicates statistically significant difference from control (one-way ANOVA, P < 0.05).

Changes in the expression levels of the anti-apoptotic protein Bcl-2 and the pro-apoptotic protein Bax were measured to investigate the mechanisms of apoptosis induction in MDA-MB-231 cells by methanolic extracts. As shown in Fig 12, the expression of Bax was increased after treatment with methanolic extract. The methanolic extract increased Bax expression levels in a time-dependent manner. The expression of Bax protein increased as early as 2 h after incubation with the methanolic extract, which was higher than control (0 h). In contrast, the expression level of Bcl-2 protein was decreased by treatment with the methanolic extract in a time dependent manner (Fig 13). The Bcl-2 protein expression decreased throughout the treatment period. As reported earlier, treatment with *S*. *ferruginea* methanol extract induced apoptosis in MDA-MB-231 cells that can be explained by an increase in Bax expression level. It can be suggested that the up-regulation of Bax protein sensitizes the MDA-MB-231 cells to apoptosis. The time-dependent down-regulation of Bcl-2 and up-regulation of Bax provides a strong indication that the mechanisms of *S*. *ferruginea*-induced apoptosis in MDA-MB-231 cells involve the intrinsic apoptosis pathway.

### Effect of methanolic extract on Caspase-3, Caspase-7, and PARP

**Fig 14:**
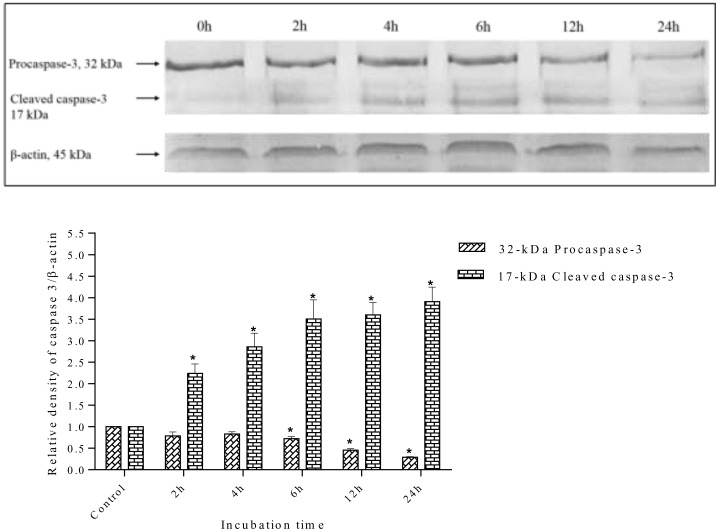
Western blot analysis of caspase-3 protein in MDA-MB-231 cells. MDA-MB-231 cells were treated with *S*. *ferruginea* methanol extract at IC_50_ for indicated times. Control cells were treated with 0.1% DMSO. β-Actin was used as loading control. Densitometric analysis demonstrated that procaspase-3 (32-kDa) was cleaved to yield a catalytically active 17-kDa fragment after treatment with the methanolic extract. The densitometric-intensity data are presented as means ± SEM of triplicate in three independent experiments. * indicates statistically significant difference from control (one-way ANOVA, P < 0.05).

**Fig 15:**
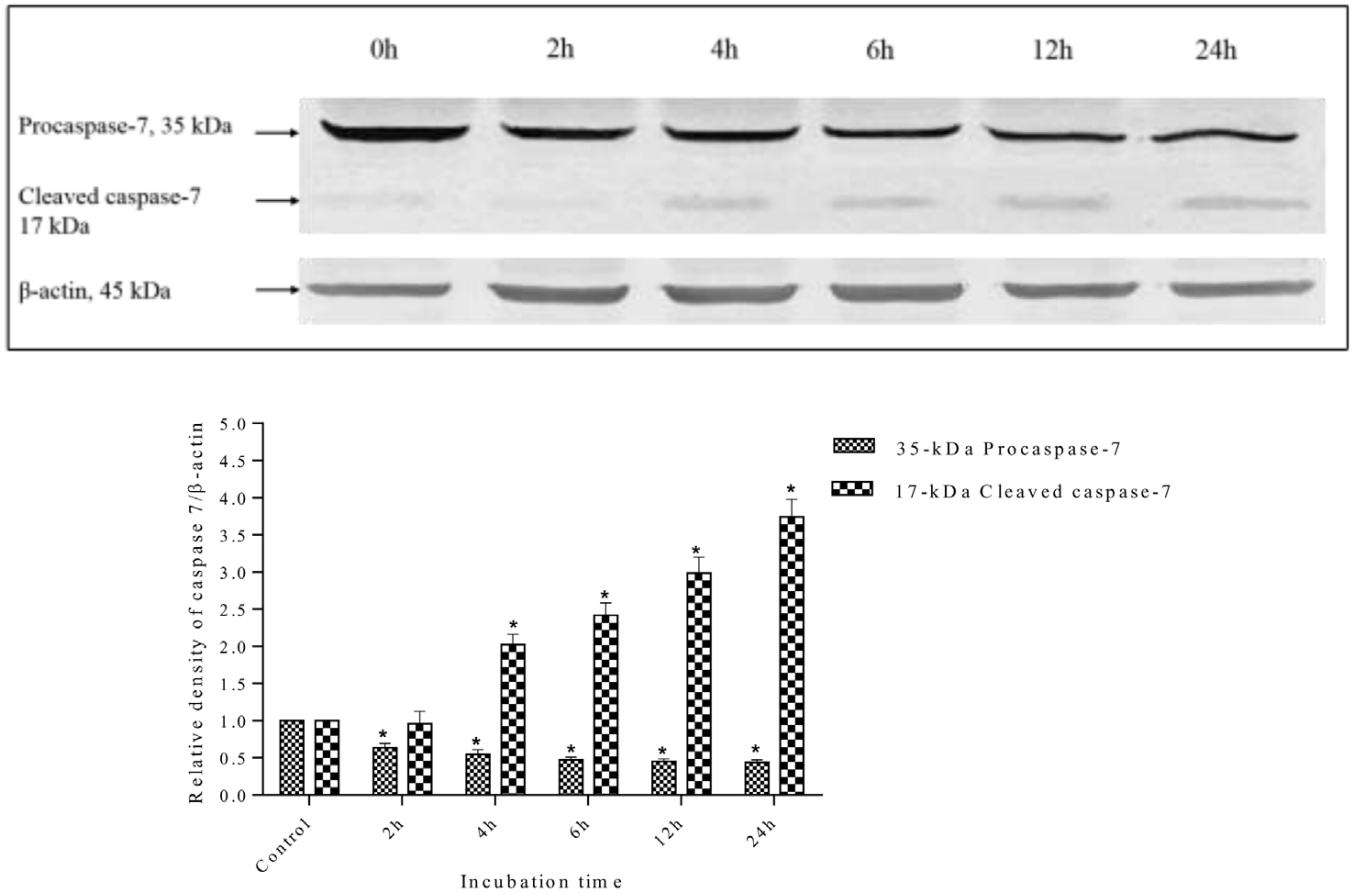
Western blot analysis of caspase-7 protein in MDA-MB-231 cells. MDA-MB-231 cells were treated with *S*. *ferruginea* methanolic extract at IC_50_ for indicated times. Control cells were treated with0.1% DMSO. β-Actin was used as loading control. Densitometric analysis demonstrated that procaspase-7 (35-kDa) was cleaved to yield a catalytically active 17-kDa fragment after treatment with methanolic extract. The densitometric-intensity data are presented as means ± SEM of triplicate in three independent experiments. * indicates statistically significant difference from control (one-way ANOVA, P < 0.05).

Caspase activation is a central requirement for apoptosis induction in cancer cells. Elucidation of the caspase activation signaling pathway activated by anticancer drugs may provide important information to design better strategies for cancer treatment. Therefore, the possible apoptosis pathways by which *S*. *ferruginea* methanolic extract induced cell death in MDA-MD-231 cells were further investigated by measuring the expression level of effector caspases (caspase-3 and caspase-7) and PARP (poly-ADP ribose polymerase). The results showed that *S*. *ferruginea* methanolic extract induced cleavage of procaspase-3 and procaspase-7 to their active forms in a time-dependent manner, as shown in Figs 14 and 15, respectively. The extract-treated groups displayed prominent reduction of procaspase-3 and procaspase-7.

**Fig 16:**
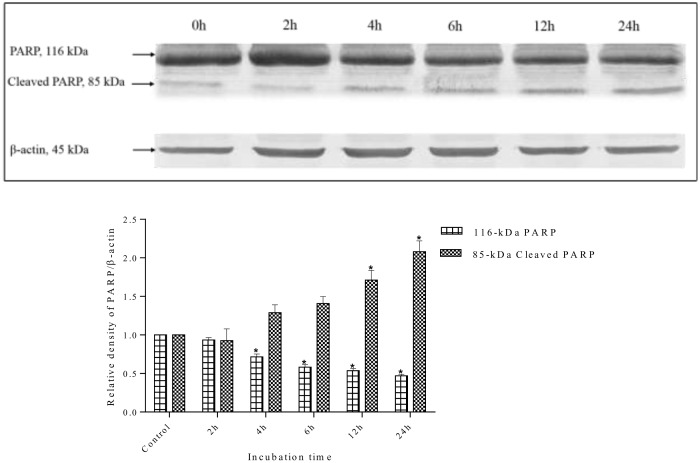
Western blot analysis of PARP protein in MDA-MB-231 cells. MDA-MB-231 cells were treated with *S*. *ferruginea* methanolic extract at IC_50_ for indicated times. Control cells were treated with 0.1% DMSO. β-Actin was used as loading control. The PARP protein (116-kDa) was cleaved into its signature 85-kDa fragment, a marker of apoptosis, after treatment with the methanolic extract. The densitometric-intensity data are presented as means ± SEM of triplicate in three independent experiments. * indicates statistically significant difference from control (one-way ANOVA, P < 0.05).

Caspase-3 is the main regulator of the final step of apoptosis that leads to proteolysis of the nuclear caspase-3 substrate, PARP. Activation of caspase-3 and caspase-7 induces the proteolytic cleavage of PARP to its signature 85-kDa fragment. Therefore, the degradation of PARP in treated cells was examined to confirm whether caspase-3 and caspase-7 are activated. Following treatment with methanol extract, the level of PARP (116-kDa) decreased, whereas the level of the cleaved 85-kDa fragment increased. This indicated that MDA-MB-231 went through apoptosis as PARP cleavage is a biochemical marker and indicator of apoptosis (Fig 16). In contrast, untreated cells at 0 h showed an intense 116-kDa band corresponding to uncleaved PARP.

With an increase in incubation time, particularly after 4 h incubation, a significant drop in 116-kDa PARP levels were observed. The level of cleaved PARP increased in a time-dependent manner in extract-treated cells. The results showed that PARP could be a downstream target in the *S*. *ferruginea* induced apoptosis pathway.

## Discussion

The present study reports the *in vitro* antioxidant capacities of the aqueous, methanolic, ethyl acetate, and hexane extracts of different *S*. *ferruginea* parts, for the first time. Stems, leaves, and flowers of S. *ferruginea* were extracted sequentially with different solvents. The methanolic extracts exhibited the highest yield. The results of this study showed that nature and polarity of solvent affect the percentage yield of the extract. This was in agreement with the study by Osadebe et al. [45], who reported a high yield of Eastern Nigeria mistletoe *(Loranthus micranthus*) extracts when using polar solvents. Previous studies on other plant species also showed the yield of samples obtained in descending order of methanol > aqueous > ethyl acetate > hexane, which corroborate results of this study. The extraction yield with methanol and water was higher than that of the other solvents [46, 47]. Furthermore, results of the current study showed that different *S*. *ferruginea* extracts contained different levels of TPC and TFC. The significant TPC and TFC differences may be attributed to different parts of plant used for extraction. The stem methanolic extract had significantly higher phenolic content than other parts (P < 0.05). Results of this study were in agreement with a previous study [48], which showed that methanolic extract of European mistletoe (*Viscum album*) were rich in phenolic compounds. The presence of large amounts of phenolic compounds in the aqueous and methanolic extracts may contribute to the antioxidant activities and the ability to adsorb and scavenge free radicals [49, 50]. TFC obtained in the present study is in line with results of Dashora et al. [51], who stated that the stem methanolic extract of Indian mistletoe (*Dendrophthoe falcata*) had higher flavonoid content than the stem aqueous extract.

Different antioxidant assays were used to evaluate the antioxidant capacities of extracts because the use of more than one assay would give a better insight into the antioxidant activity of the extracts. Generally, the antioxidant potential of plant extracts is associated with the method of extraction, polarity of extraction solvent, composition of the extract, and conditions of the test systems. Results of the present study showed that the different *S*. *ferruginea* extracts possessed diverse antioxidant abilities. The differences in antioxidant activity may be attributed to different extraction solvents and plant parts used in the study. Generally, the crude methanolic and aqueous extracts had higher antioxidant potential than the corresponding ethyl acetate and hexane extracts. Furthermore, the methanolic extracts exhibited higher antioxidant activity than the aqueous extracts. Similar results were obtained previously with the methanolic extract of Indian mistletoe *Dendrophthoe falcate* (IC_50_ = 18 μg/mL), which showed higher scavenging activity than the aqueous extract (IC_50_ = 26 μg/mL) [51]. Results of this study can also be compared with the results of [52], which showed that the stem methanolic extract of *Loranthus europaeus* showed higher DPPH^•^ inhibition (88%) than the corresponding flower (79%) and leaf methanol extracts (86%). In addition, ABTS radical cation assay results of the present study are consistent with those reported by Vicaş et al. [53], who showed that the aqueous leaf and stem extracts of *V*. *album* had the highest level of ABTS^•+^ scavenging activity. Results of the radical scavenging activity clearly demonstrated that the type of extractant and solvent polarity markedly influence the antioxidant activities, TPC, and TFC of *S*. *ferruginea* extracts. In recent years, extensive studies have focused on the antioxidant activity of many mistletoe species growing on different host trees [52, 54–56]. Mistletoe extracts exhibited various degrees of radical scavenging capacity depending on the harvesting time, host tree, and geographical location. Onay-Uçar et al. [54] evaluated the radical scavenging activity of crude extracts from European mistletoe (*V*. *album*) growing on different host trees by DPPH method. They showed that the methanol extract of *V*. *album* growing on lemon tree exhibited the highest scavenging capacity (95.12 ± 2.37%), even higher than standards (BHA and vitamin E). They also demonstrated that different scavenging abilities of the extracts are attributable to the differences in the nature of host trees as well as harvesting time. An investigation on aqueous extracts of *V*. *album* from 5 different host trees [55] proved the influence of harvesting time and host tree on the antioxidant ability. The aqueous extract of *Robinia pseudocacia* showed the highest radical scavenging capacity.

Chelating agents are effective as secondary antioxidants since they are capable of stabilizing the oxidized form of the metal ion by reducing the redox potential [57]. A study by A. Oluwaseun and O. Ganiyu showed the significant and dose-dependent iron chelating capacity of *V*. *album* from cashew tree [6]. The Pearson correlation analysis revealed a highly positive correlation between TPC and antioxidant activity assays indicating that phenolic compounds were the major contributor to the antioxidant capacity of the *S*. *ferruginea* extracts. These results were in accordance with a previous study [48], which reported a significant correlation between TPC and antioxidant activity (determined by FRAP assay for leaves and stems extracts of *Viscum album*). However, in a subsequent study [53], the same author demonstrated that there was no correlation between antioxidant potential TPC of *V*. *album* from different host trees determined by DPPH, ORAC, and TEAC methods. Collectively, stem methanolic extract, which contained the highest amount of phenolic and flavonoid compounds, showed strong DPPH^•^ scavenging capacity, and pronounced metal chelation activity. However, stem aqueous extract exhibited the highest scavenging ability on ABTS^•+^ compared to other extracts.

Stem methanolic and aqueous extracts showed high antioxidant activities, TPC, and TFC. Their cytotoxic effects were further investigated on human breast cancer cell line, MDA-MB-231, and non-cancer human skin fibroblast cell line, HSF-1184. To the best of our knowledge, this study appears to be the first report on the cytotoxic activity of *S*. *ferruginea* extracts against human breast cancer cell line, MDA-MB-231. MTT assay showed that extracts exhibited anti-proliferative effects on cancer cells in a time-and dose-dependent manner. In contrast, the extracts did not significantly affect the viability of normal cells. Stem methanolic and aqueous extracts showed cytotoxic activity towards breast cancer cells without showing toxicity to normal cells even at high concentrations. Currently, anti-cancer agents with ability to induce apoptosis in tumor cells without exerting cytotoxic effects on non-malignant cells have gained attention of scientists for developing new cancer chemotherapeutics [58]. According to the United States National Cancer Institute (NCI) plant screening program, a crude extract is considered to have an active *in vitro* cytotoxic activity if its IC_50_ following incubation between 48 and 72 h is less than 20 μg/mL [59, 60]. Based on this threshold, stem methanolic extract in this study possessed potential cytotoxic activity. This appears to be contrary to the findings of Dashora et al. [51], who reported that the cytotoxic effect of aqueous extract (IC_50_ = 90 μg/mL) was higher than the ethanol extract. However, it is noteworthy that they used ethanol and water for extraction, while the present study used methanol and water.

It has been shown that the phenolic compounds, particularly at high concentrations, can become pro-oxidants and change the redox balance of cancer cells [61]. The potential cytotoxic effect found in the present study might be attributed to the phenolic compounds present in the methanolic extract of *S*. *ferruginea* stem. This extract showed the highest TPC and causes cell death at the lowest concentration.

The mode of cell death induced by *S*. *ferruginea* extracts was examined through morphological changes in cells. Morphological alterations in apoptotic cells are the most important means for apoptosis detection [62]. MDA-MB-231 cells treated with selected extracts revealed hallmark properties of apoptosis including cell shrinkage, cell rounding, and cytoplasmic condensation, as shown by light microscopy. These morphological alterations were previously reported in apoptotic cells [63–65]. Consistent with the morphological alterations, fluorescence microscopy also demonstrated pro-apoptotic effects of extracts. Methanolic and aqueous extracts altered cellular morphology of MDA-MB-231 cells in a dose dependent manner, which was also observed by AO/EB staining. The induction of apoptosis was further confirmed by Hoechst 33342/PI staining assay.

More than 90% of human cancer-related deaths occur due to metastatic spread of cancer cells, which remains one of the biggest obstacles in cancer treatment [66, 67]. Cell migration is one of the most crucial steps involved in metastasis [68]. The most dangerous and life-threatening stage of cancer is metastasis of cancer cells to vital organs. Therefore, targeting highly metastatic cancer cells is critical for development of novel chemotherapeutic anticancer drugs. MDA-MB-231 cell line is a rapidly proliferating breast cancer cell line. It is a well-established model to evaluate the anti-metastatic effects of therapeutic compounds and to investigate the mechanism of cancer metastasis [69, 70]. Findings of this study showed that the stem aqueous and methanolic extracts at various concentrations, particularly at toxic doses, inhibited the migration of MDA-MB-231 cells. It can be suggested that the inhibitory effects of extracts on MDA-MB-231 cell-migration were likely due to its cytotoxic effect. Antiproliferative activity of *S*. *ferruginea* extracts was further confirmed by colony forming assay. Selected extracts (in particular methanolic extract) suppressed colony formation of MDA-MB-231 cells in a dose-dependent manner. Pair-wise comparison of the methanolic versus aqueous extract shows that the latter is less effective in suppressing the growth, migration, and colony-forming abilities of MDA-MB-231 cell line. Therefore, further mechanistic studies were carried out on methanolic extract. The elevated level of ROS generation in mitochondria disrupts MMP [71] and subsequently leads to the release of cytochrome c that activates caspase to initiate apoptosis [72]. Therefore, the present study determined whether the apoptosis induced by methanolic extract is through mitochondrial mediated pathway. The level of ROS generation was determined by the fluorescent dye DCF-DA, which is readily oxidized to 2',7'-dichlorofluorescein (DCF) in presence of ROS. Results revealed that *S*. *ferruginea* stem methanolic extract induced mitochondrial mediated apoptosis in MDA-MB-231. It is in agreement with previous findings that showed an increased ROS generation in cancer cells after treatment with plant extracts [73, 74]. Generally, early apoptosis is accompanied by reduction or loss of MMP, resulting in a rapid collapse of the electrochemical gradient across the mitochondrial membrane [75]. The MMP (*ΔΨ*m) was measured by JC-1 assay. A concentration-dependent depolarization of mitochondrial membrane was observed in cells treated with methanolic extract. The JC-1 assay results were consistent with those obtained from ROS generation, indicating that methanolic extract altered the MMP in breast cancer cells, which suggested its potent apoptotic activity.

Targeting the molecular pathways involved in apoptosis of cancer cells plays a crucial role in development of anticancer drug for treatment of cancer [76]. Therefore, Western blot analysis was carried out to identify molecular compounds that induce apoptosis in MDA-MB-231 cells after treatment with *S*. *ferruginea* methanolic extract.

Bcl-2 family members (Bcl-2 and Bax) are key regulators of the intrinsic apoptotic pathway and have the responsibility of cell death or cell survival regulation in different situations [77]. Western blot analysis showed that methanolic extract increased level of Bax protein and reduced Bcl-2 protein level. The elevated Bax to Bcl-2 ratio could lead to loss in MMP that affects the release of cytochrome c from the mitochondria and activates the caspase cascade [78]. Subsequently, proteolytic cleavage of both procaspase-3 and procaspase-7 to their active form was examined. Activated caspase-3 and caspase-7 cleave PARP, which consequently promotes DNA fragmentation and cell shrinkage, resulting in apoptosis induction [79–80].

## Conclusion

The *S*. *ferruginea* stem methanolic extract induced cytotoxicity and apoptosis in MDA-MB-231 cells. It also induced ROS generation and disrupted the MMP. In addition, this study demonstrated that the *S*. *ferruginea* methanolic extract induced apoptosis possibly through intrinsic apoptotic signaling pathway. Antiproliferative and apoptotic effects of methanolic extract against MDA-MB-231 cells could be attributed to their antioxidant activity, high phenolic and flavonoid contents. However, specific bioactive compounds responsible for the anti-breast cancer activity of extracts require attention. Further detailed studies on the underlying mechanism responsible for the antioxidant and anticancer activities are needed. Taken together, the findings of this study showed antioxidant and anticancer activities of *S*. *ferruginea* extracts and provided the scientific rationale for using this plant in future development of chemotherapeutic drugs against breast cancer.
